# Net ultrafiltration prescription survey in Europe

**DOI:** 10.1186/s12882-020-02184-y

**Published:** 2020-12-01

**Authors:** Nuttha Lumlertgul, Raghavan Murugan, Nina Seylanova, Patricia McCready, Marlies Ostermann

**Affiliations:** 1 Department of Critical Care, King’s College London, Guy’s & St Thomas’ Hospital, NHS Foundation Trust, London, SE1 7EH UK; 2 Division of Nephrology, Department of Internal medicine, King Chulalongkorn Memorial Hospital, Bangkok, Thailand; 3 Excellence Center in Critical Care Nephrology, King Chulalongkorn Memorial Hospital, Bangkok, Thailand; 4 Research Unit in Critical Care Nephrology, Chulalongkorn University, Bangkok, Thailand; 5 The Center for Critical Care Nephrology, Department of Critical Care Medicine, University of Pittsburgh School of Medicine, Pittsburgh, PA USA; 6 The Clinical Research, Investigation, and Systems Modeling of Acute Illness (CRISMA) Center, Department of Critical Care Medicine, University of Pittsburgh School of Medicine, Pittsburgh, PA USA; 7 Sechenov Biomedical Science and Technology Park, Sechenov First Moscow State Medical University, Moscow, Russian Federation

**Keywords:** Fluid overload, Ultrafiltration, Renal replacement therapy, Fluid removal

## Abstract

**Background:**

Fluid overload is common in patients in the intensive care unit (ICU) and ultrafiltration (UF) is frequently required. There is lack of guidance on optimal UF practice. We aimed to explore patterns of UF practice, barriers to achieving UF targets, and concerns related to UF practice among practitioners working in Europe.

**Methods:**

This was a sub-study of an international open survey with focus on adult intensivists and nephrologists, advanced practice providers, and ICU and dialysis nurses working in Europe.

**Results:**

Four hundred eighty-five practitioners (75% intensivists) from 31 countries completed the survey. The most common criteria for UF initiation was persistent oliguria/anuria (45.6%), followed by pulmonary edema (16.7%). Continuous renal replacement therapy was the preferred initial modality (90.0%). The median initial and maximal rate of net ultrafiltration (UF^NET^) prescription in hemodynamically stable patients were 149 mL/hr. (IQR 100–200) and 300 mL/hr. (IQR 201–352), respectively, compared to a median UF^NET^ rate of 98 mL/hr. (IQR 51–108) in hemodynamically unstable patients and varied significantly between countries.

Two-thirds of nurses and 15.5% of physicians reported assessing fluid balance hourly. When hemodynamic instability occurred, 70.1% of practitioners reported decreasing the rate of fluid removal, followed by starting or increasing the dose of a vasopressor (51.3%). Most respondents (90.7%) believed in early fluid removal and expressed willingness to participate in a study comparing protocol-based fluid removal versus usual care.

**Conclusions:**

There was a significant variation in UF practice and perception among practitioners in Europe. Future research should focus on identifying the best strategies of prescribing and managing ultrafiltration in critically ill patients.

**Supplementary Information:**

The online version contains supplementary material available at 10.1186/s12882-020-02184-y.

## Background

Fluid overload is common in intensive care units (ICU) and is strongly associated with increased mortality, impaired renal recovery, and distant organ dysfunction among critically ill patients [1, 2]. Achieving euvolemia after the initial fluid resuscitation phase consists of minimizing fluid input and removing excessive fluid [3]. There are two strategies for removing fluid, diuretic pharmacotherapy and mechanical fluid removal using slow continuous ultrafiltration (SCUF) or renal replacement therapy (RRT) [4]. Mechanical fluid removal is usually considered in patients deemed inadequately responsive to diuretics, so called ‘diuretic-resistant’ [4, 5]. However, there is no clear definition of diuretic resistance, and consensus criteria for the practice of ultrafiltration including the indications, the timing of initiation, optimal dosing, and monitoring are lacking.

Fluid accumulation is common in patients with acute kidney injury (AKI) [6, 7]. Although there are many studies exploring different aspects of RRT, including dose, modality, and timing, only few have investigated the management of fluid removal, monitoring, and complication management of ultrafiltration [8–13]. In 2016, the Acute Disease Quality Initiative (ADQI) Consensus Group published recommendations for management of fluid balance and regulation during RRT [14]. Several areas of uncertainty were acknowledged.

Recently, we reported the results of a multinational survey of critical care practitioners and demonstrated that there was significant variation in practice worldwide [15]. The aim of this sub-study was to get more insight into clinical practice among clinicians working in hospitals in Europe, in particular, the criteria for initiation of ultrafiltration, prescription, monitoring of fluid balance, management of complications, and perceived barriers to successful fluid removal. We also explored the attitudes of practitioners towards protocol-based management, and willingness to enroll patients in clinical trials comparing protocol-based ultrafiltration versus usual care.

## Methods

### Survey administration

We developed a 25-question survey, which was approved by the University of Pittsburgh’s Human Research Protection Office and endorsed by the European Society of Intensive Care Medicine (ESICM), the National Institute of Health Research in the United Kingdom and the respective national approval committees [Supplementary Content 1] [15]. In Europe, the survey was distributed to adult intensivists and nephrologists including trainees, advanced practice providers (i.e., nurse practitioners), and ICU and dialysis nurses via the British Association of Critical Care Nurses (BACCN), ESICM, and Italian Society of Intensive Care between January 6, 2018, and January 10, 2019. Reminder emails were sent by each society according to their policies, and links to the survey were displayed on their websites and in the BACCN newsletter. In addition, the investigators also sent links to the survey to their professional networks, for instance the London AKI Network in London, UK. Participation was voluntary and fully anonymized. No identifiable data were collected. Consent was implied upon the completion of the survey. We adhered to the Checklist for Reporting Results of Internet E-surveys to report the data.

The survey was conducted in English. All main questions were set as compulsory fields. Questions 5, 6 and 7 pertained to staff who typically prescribe diuretics and make decision on initiation and prescription of UF, therefore ICU and dialysis nurses were excluded from these questions.

### Statistical analysis

Only complete questionnaires were included in the final analyses. We present descriptive statistics as either proportions, mean (standard deviation), or median (interquartile range [IQR]), as appropriate. We assessed practice variation using the chi-square test and Wilcoxon rank sum for binary outcomes, and t-test with unequal variances or Kruskal-Wallis test for continuous outcomes, respectively. We did not impute any missing data and considered *p* values less than 0.05 to be statistically significant. All analyses were performed using STATA 14.1 (STATA Corp, College Station, TX) software. Thematic analysis was performed of free text comments.

## Results

### Practitioner characteristics

**Fig. 1:**
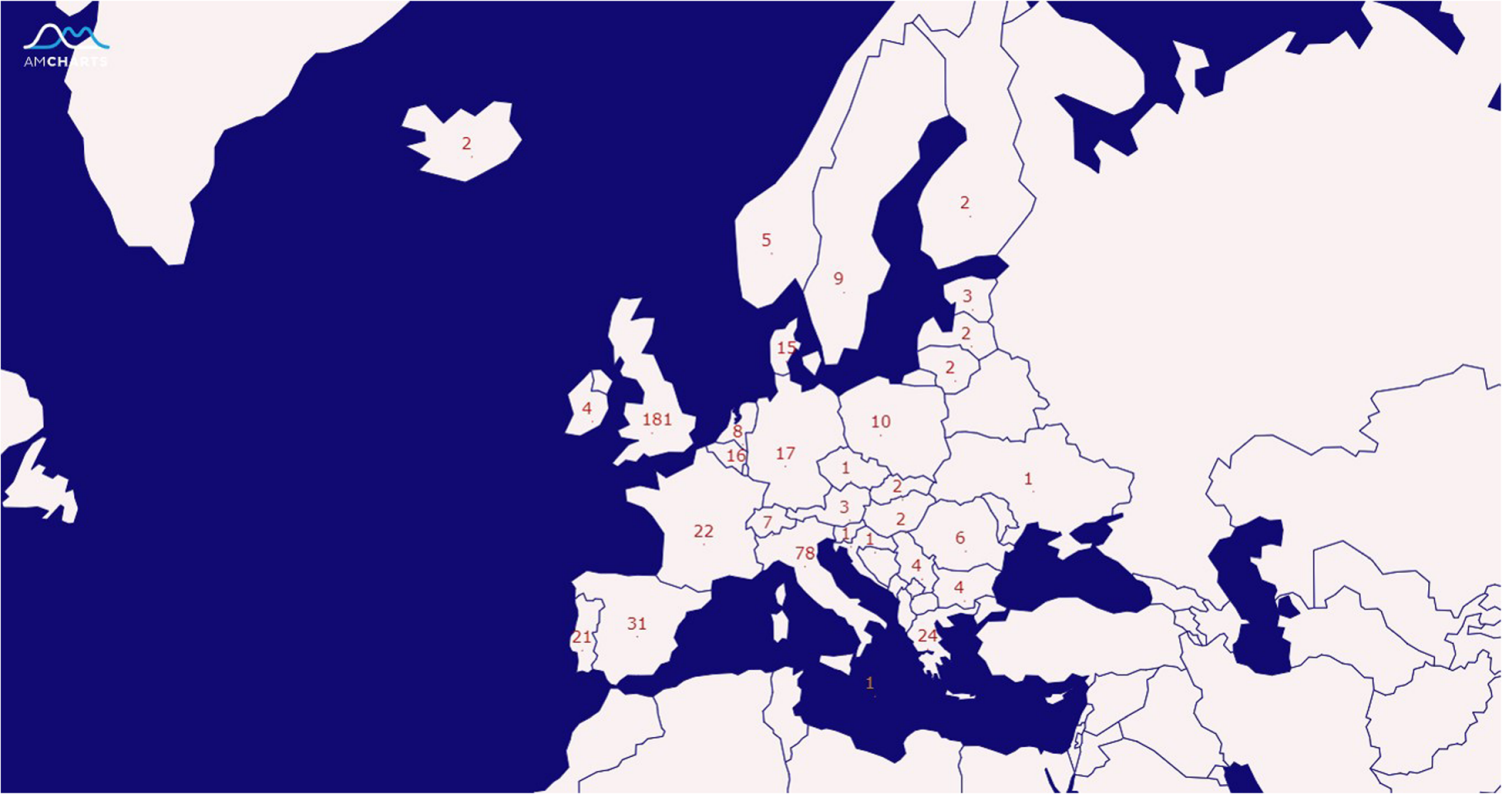
Number of participants by countries [Adapted with permission from [16]]. The number on each country’s map represents the number of practitioners per country who completed the survey. Total numbers of participants who completed the survey was 485. The figure was generated with http://pixelmap.amcharts.com with permission from amCharts

The survey was distributed to 23,009 practitioners from three societies (16,360 from ESICM, 4649 from Italian Society of Intensive care, and 2000 from BACCN). There were 679 practitioners from 31 European countries who responded of whom 485 (71.4%) completed the entire questionnaire. The most represented countries were the United Kingdom (UK) (37.3%), Italy (16.1%), Spain (6.4%), Greece (5.0%), France (4.5%), Portugal (4.3%), and Germany (3.5%); Fig. 1. Approximately 75% were intensivists and 18.3% were ICU nurses. The median duration of clinical experience was 16.3 years (IQR, 10–23.9 years). Physicians who responded had relatively more years of clinical experience than nurses (18.0; IQR 11.0–25.0 vs 10.0; IQR 6.0–19.4 years; *p* < 0.001) (Supplementary data: Table S1). About two-thirds (63.1%) practiced in university-based hospitals, 22.7% in community hospitals, and 8.5% in government hospitals. There was a higher proportion of physicians working in community hospitals (26.6% vs 7.2%), and more nurses from government hospitals than physicians (22.7% vs 4.9%). Most practitioners (70.6%) with less than 15 years of experience practiced in university-based hospitals (Supplementary data: Table S2).

### Diuretic resistance and criteria used for ultrafiltration

The majority of responses related to prescribing of diuretics, criteria for UF initiation, and UF prescription stemmed from physicians (Table 1). High dose diuretics were commonly prescribed for fluid management. About one-third (33.6%) of physicians used a maximum 100–250 mg of furosemide equivalent per day before determining diuretic resistance, followed by 17.5% who prescribed 251–500 mg/d. (Supplementary data: Figure S1) Key triggers for initiation of ultrafiltration were persistent oliguria/anuria (45.6%), pulmonary edema (16.7%), and severe hypoxemia (9.6%). When determining targets for net ultrafiltration (UF^NET^), practitioners based the decision on hemodynamic status (44.8%), cumulative fluid balance (22.3%), and 24-h fluid balance (15.9%) (Table 1).

**Table 1:** Comparison between doctors and nurses/nurse practitioners

Characteristic	All (***n*** = 485)	No. (%)		***P*** Value
		Doctors (***n*** = 388)	Nurses and nurse practitioners (***n*** = 97)	
Country				
United Kingdom	181 (37.3)	101 (55.8)	80 (44.2)	**< 0.001**
Italy	78 (16.1)	68 (87.2)	10 (12.8)	
Spain	31 (6.4)	31 (100.0)	0	
Greece	24 (5.0)	24 (100.0)	0	
France	22 (4.5)	22 (100.0)	0	
Portugal	21 (4.3)	19 (90.5)	2 (9.5)	
Germany	17 (3.5)	17 (100.0)	0	
Others	111 (22.9)	106 (95.5)	5 (4.5)	
Occupation				
Advanced practice provider	7 (1.4)			
Dialysis nurse	1 (0.2)			
ICU nurse	89 (18.4)	–	–	
Intensivist	365 (75.3)			
Intensivist and nephrologist	19 (3.9)			
Nephrologist	4 (0.8)			
Years of practice, median (IQR)	16.3 (10–23.9)	18.0 (11.0–25.0)	10.0 (6.0–19.4)	**< 0.001**
Hospital Type				
University-based	306 (63.1)	247 (63.7)	59 (60.8)	**< 0.001**
Community-based	110 (22.7)	103 (26.6)	7 (7.2)	
Government	41 (8.5)	19 (4.9)	22 (22.7)	
Other	28 (5.7)	19 (4.9)	9 (9.3)	
Maximum dose of loop diuretic prescribed (furosemide equivalent)^a^, mgs/day (*n* = 394)				
< 100	41 (10.4)	41 (10.6)	0 (0.0)	0.32
100–250	132 (33.5)	130 (33.6)	2 (28.6)	
251–500	69 (17.5)	68 (17.6)	1 (14.3)	
501–750	25 (6.4)	24 (6.2)	1 (14.3)	
751–1000	66 (16.8)	65 (16.8)	1 (14.3)	
> 1000	30 (7.6)	30 (7.8)	0 (0.0)	
Other dose (e.g. 1–1.5 mg/kg)	8 (2.0)	8 (2.1)	0 (0.0)	
I do not prescribe diuretics.	23 (5.8)	21 (5.4)	2 (28.6)	
Criteria used for initiation of UF ^a^ (*n* = 395)				
Persistent oliguria/anuria (urine output < 0.5 mL/kg/hour for ≥12 h)	180 (45.6)	176 (45.4)	4 (57.1)	0.23
Severe hypoxemia (PaO_2_/FiO_2_ ratio < 150)	38 (9.6)	38 (9.8)	0 (0.0)	
Pulmonary edema with or without hypoxemia	66 (16.7)	65 (16.8)	1 (14.3)	
Cumulative fluid balance (> 1000 mL)	19 (4.8)	18 (4.6)	1 (14.3)	
Fluid overload > 10% of body weight	21 (5.3)	21 (5.4)	0 (0.0)	
Ongoing need for fluids in the presence of oliguria	26 (6.6)	26 (6.7)	0 (0.0)	
I do not make the decision	5 (1.3)	4 (1.0)	1 (14.3)	
I use other criteria (e.g. acidosis, hyperkalemia, uremia) or combination of above criteria	40 (10.1)	40 (10.3)	0	
Criteria used for prescription of UF^NET a^ (n = 395)				
24-h fluid balance	63 (15.9)	62 (16.0)	1 (14.3)	0.12
Cumulative fluid balance	88 (22.3)	86 (22.2)	2 (28.6)	
Weight gain	31 (7.9)	31 (8.0)	0 (0.0)	
Radiographic features of fluid overload	7 (1.8)	7 (1.8)	0 (0.0)	
Hemodynamic status (HR, BP, CVP, PPV, dose of vasopressors)	177 (44.8)	175 (45.1)	2 (28.6)	
Volume of anticipated fluid use in the next 24 h	10 (2.5)	10 (2.6)	0 (0.0)	
Arterial lactate	1 (0.3)	1 (0.3)	0 (0.0)	
I do not prescribe UF.	3 (0.8)	2 (0.5)	1 (14.3)	
Others e.g. more than one criteria, lung ultrasound	15 (3.8)	14 (3.6)	1 (14.3)	
IHD use, median (IQR)				
Percent use last month	5.0 (0–25.0)	5.0 (0.0–21.0)	5.0 (0.5–32.5)	0.18
Typical prescription, mL per session	2000 (1500–3000)	2000 (1500–3000)	2000 (1900–3000)	0.91
Slow forms of IHD use, median (IQR)				
Percent use last month	1.0 (0–20.0)	1.0 (0–18.0)	1.0 (0–20.0)	0.87
Typical prescription, mL per session	2000 (1000–2000)	2000 (1000–2900)	3000 (0–4000)	0.55
Percent of assessment of prescription-to-delivered UF^NET^, median (IQR)	79.5 (21.0–100.0)	74.0 (28.0–100.0)	81.0 (10.0–100.0)	0.92
CRRT use, median (IQR)				
Percent use in the last month	90.0 (30.0–100.0)	90.0 (30.0–100.0)	82.5 (41.5–100.0)	0.60
Initial UF rate for hemodynamically stable patient, mL per hour	149.0 (100.0–200.0)	151 (100–200)	102 (100–200)	0.058
Maximal UF rate for hemodynamically stable patient, mL per hour	300.0 (201.0–352.0)	300 (201–358)	300 (248–351)	0.83
UF rate for hemodynamically unstable patient, mL per hour	98.0 (51.0–108.0)	98 (51–106)	81 (51–120)	0.78
Method used to achieve UF using CRRT, No. (%) (*n* = 463)				
varying ultrafiltration rate only	191 (41.3)	133 (36.1)	58 (61.1)	**< 0.001**
varying replacement fluid rate only	32 (6.9)	30 (8.2)	2 (2.1)	
varying both ultrafiltration and replacement fluid rate	191 (41.3)	166 (45.1)	25 (26.3)	
I do not know.	36 (7.8)	29 (7.9)	7 (7.4)	
I do not prescribe UF.	13 (2.8)	10 (2.7)	3 (3.2)	
How frequently do you check net fluid balance during CRRT? No. (%) (n = 463)				
1 h	121 (26.1)	57 (15.5)	64 (67.4)	**< 0.001**
2 h	20 (4.3)	16 (4.4)	4 (4.2)	
4 h	40 (8.6)	35 (9.5)	5 (5.3)	
6 h	57 (12.3)	51 (13.9)	6 (6.3)	
8 h	63 (13.6)	57 (15.5)	6 (6.3)	
12 h	67 (14.5)	65 (17.7)	2 (2.1)	
24 h	55 (11.9)	53 (14.4)	2 (2.1)	
I do not check net fluid balance.	40 (8.6)	34 (9.2)	6 (6.3)	
Percentage of patients developing new hemodynamic instability during UF, median (IQR)	20.0 (10.0–30.0)	20.0 (10.0–30.0)	14.0 (5.0–30.0)	0.20
Interventions performed for hemodynamic instability				
Decrease the rate of fluid removal	341 (70.1)	269 (69.3)	72 (74.2)	0.35
Completely stop fluid removal	165 (33.8)	119 (30.7)	46 (47.4)	**0.002**
Make no changes to fluid removal rate	19 (3.7)	15 (3.9)	4 (4.1)	0.91
Administer a fluid bolus	175 (36.5)	125 (32.2)	50 (51.6)	**< 0.001**
Start or increase the dose of a vasopressor	245 (51.3)	187 (48.2)	58 (59.8)	**0.041**
Switch to alternative modality	16 (3.3)	14 (3.6)	2 (2.1)	0.45
Administer albumin or mannitol bolus	61 (13.4)	50 (12.9)	11 (11.3)	0.68
Perceived barriers to UF^NET^				
Patient intolerance (e.g.*,* hypotension)	354 (72.6)	271 (69.9)	83 (85.6)	**0.002**
Under prescription	71 (15.2)	66 (17.0)	5 (5.2)	**0.003**
Frequent interruptions (e.g.*,* trip to CT scan, operating room, filter clotting, catheter malfunction)	221 (45.3)	158 (40.7)	63 (65.0)	**< 0.001**
Inability to titrate fluid removal	21 (4.5)	14 (3.6)	7 (7.2)	0.12
Unavailability of adequately trained nursing staff	37 (7.4)	31 (8.0)	6 (6.2)	0.55
Unavailability of dialysis machines	29 (6.2)	24 (6.2)	5 (5.2)	0.70
Cost associated with treatment	23 (4.7)	19 (4.9)	4 (4.1)	0.75
I believe early fluid removal is beneficial				
Strongly agree	159 (32.8)	127 (32.7)	32 (33.0)	0.65
Agree	195 (40.2)	152 (39.2)	43 (44.3)	
Somewhat agree	86 (17.7)	71 (18.3)	15 (15.5)	
Neither agree nor disagree	34 (7.0)	28 (7.2)	6 (6.2)	
Somewhat disagree	8 (1.7)	8 (2.1)	0	
Disagree	3 (0.6)	2 (0.5)	1 (1.0)	
I believe a protocol-based fluid removal strategy would be beneficial				
Strongly agree	123 (25.4)	99 (25.5)	24 (24.7)	0.13
Agree	148 (30.5)	122 (31.4)	26 (26.8)	
Somewhat agree	103 (21.2)	81 (20.9)	22 (22.7)	
Neither agree nor disagree	52 (10.7)	44 (11.3)	8 (8.3)	
Somewhat disagree	28 (5.8)	20 (5.2)	8 (8.3)	
Disagree	22 (4.5)	18 (4.6)	4 (4.1)	
Strongly disagree	9 (1.9)	4 (1.0)	5 (5.2)	
I would enroll my patient in a clinical trial comparing protocol-based versus usual care (*n* = 484)				
Strongly agree	127 (26.2)	105 (27.1)	22 (22.9)	**0.001**
Agree	195 (40.3)	160 (41.2)	35 (36.5)	
Somewhat agree	72 (14.9)	63 (16.2)	9 (9.4)	
Neither agree nor disagree	61 (12.6)	37 (9.5)	24 (25)	
Somewhat disagree	11 (2.3)	9 (2.3)	2 (2.1)	
Disagree	15 (3.1)	13 (3.4)	2 (2.1)	
Strongly disagree	3 (0.6)	1 (0.3)	2 (2.1	

*Abbreviations*: *BP* blood pressure, *CRRT* continuous renal replacement therapy, *CT* computed tomography, *CVP* central venous pressure, *HR* heart rate, *ICU* intensive care unit, *IQR* interquartile range, *IHD* intensive care unit, *UF* ultrafiltration

^a^Practitioners included intensivists, nephrologists, intensivists and nephrologists, and advanced practice providers. ICU and dialysis nurses were excluded from these questions

Among the top seven respondent countries, practitioners from the UK, Portugal, and Germany were more likely to prescribe a maximum of 100–250 mg of diuretics, while more practitioners from Italy, Spain, Greece, and France used a maximum of 751–1000 mg (*p* < 0.001). More than half of practitioners from Italy, Spain, Greece, and Germany used persistent oliguria/anuria as a criterion for extracorporeal fluid removal, while one-third of practitioners from the UK used pulmonary edema as a trigger (*p* < 0.001) (Supplementary data: Table S3).

### Fluid removal practice

#### Modality

##### Intermittent hemodialysis

**Fig. 2:**
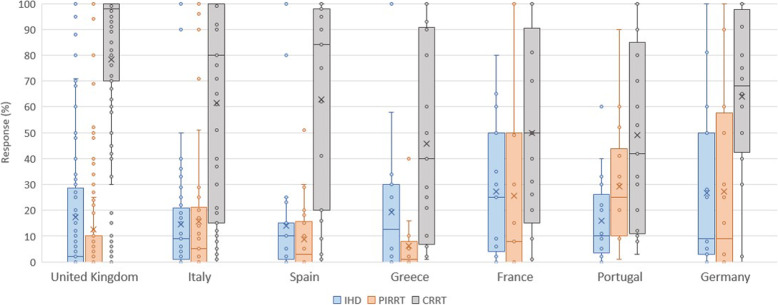
Modalities of RRT use among the top seven respondent countries. Boxplot summaries of modalities of renal replacement therapy among the top seven respondent countries. The vertical box represents the 25th percentile (bottom line), median (middle line), and 75th percentile (top line) values. The lowest datum (lower whisker) represents 1.5 times the interquartile range of the lower quartile, and the highest datum (upper whisker) represents 1.5 times the interquartile range of the upper quartile. Circles represent outliers. Prolonged intermittent renal replacement therapy (PIRRT) and continuous renal replacement therapy (CRRT) varied significantly between countries (*p* < 0.001), whereas the use of intermittent hemodialysis (IHD) was not different between different types of hospitals (*p* = 0.13)

Practitioners reported using intermittent hemodialysis (IHD) in a median of 5.0% (IQR, 0–25.0%) and prolonged intermittent RRT (PIRRT) in a median of 1.0% (IQR, 0–20%) of cases. Among the top seven respondent countries, PIRRT was more commonly used in Portugal (25.0%, IQR 10.0–41.0%) (Fig. 2). IHD was more commonly used in other types of hospitals (15.0%) compared to university-based (5%), community (9%), or government (8%) hospitals; *p* = 0.04 (Supplementary data: Table S2, Figure S2). The typical median prescribed UF^NET^ rate was 2000 mL/session (IQR, 1500–3000) for IHD and 2000 mL/session (IQR, 1000–2000) for PIRRT. Respondents from France reported prescribing higher UF^NET^ rate for PIRRT (3500 mL/session, IQR 2000–4000). Practitioners with more than 15 years of clinical practice prescribed higher UF^NET^ rates than clinicians with less experience (mean 2328 ± 1512 mL/session vs 1575 ± 1071 mL/session); *p* = 0.0006. The majority (79.5%; IQR, 21.0–100.0%) reported that they assessed prescribed-to-delivered UF^NET^ routinely, and 12.8% stated that they assessed prescribed-to-delivered UF^NET^ less than 25% of the time.

##### Continuous RRT

**Fig. 3:**
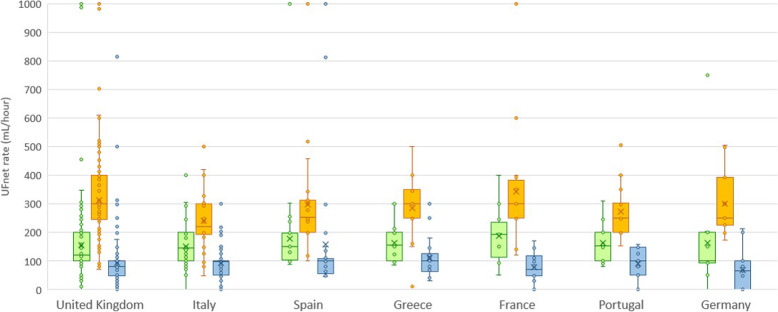
Variations in UF^NET^ prescription among Top Seven Respondent Countries. Boxplot summaries of initial and maximal net ultrafiltration rates for hemodynamically stable patients and typical net ultrafiltration rates for hemodynamically unstable patients for the top seven respondent countries. The vertical box represents the 25th percentile (bottom line), median (middle line), and 75th percentile (top line) values. The lowest datum (lower whisker) represents 1.5 times the interquartile range of the lower quartile, and the highest datum (upper whisker) represents 1.5 times the interquartile range of the upper quartile. Circles represent outliers. Net ultrafiltration rates varied significantly across countries (*p* < 0.001 for all three groups)

Most physicians (90.0%; IQR, 30.0–100.0%) stated that they used continuous RRT (CRRT) as the first modality for ultrafiltration. Practitioners in university-based hospitals were more likely to use CRRT (90.0%; IQR, 50.0–100.0%) than people working in government hospitals (72.0%; IQR, 22.5–100.0%), other types of hospital (68.0%; IQR, 10.0–100.0%), and community hospitals (66.0%; IQR, 19.0–100.0%). For hemodynamically stable patients, the median initial UF^NET^ prescription was 149.0 mL/hr. (IQR, 100.0–200.0), with variation between countries. The maximal UF^NET^ rate prescribed was 300.0 mL/hr. (IQR, 201.0–352.0). For hemodynamically unstable patients, the reported median UF^NET^ rate was 98.0 mL/hr. (IQR, 51.0–108.0) (Fig. 3 and Supplementary data: Table S4). Respondents with more than 15 years of clinical experience prescribed higher UF^NET^ rates in hemodynamically unstable patients (100 mL/hr.; IQR 51–120 vs 76 mL/hr.; IQR 49–101; *p* = 0.02) compared to those with less experience.

**Fig. 4:**
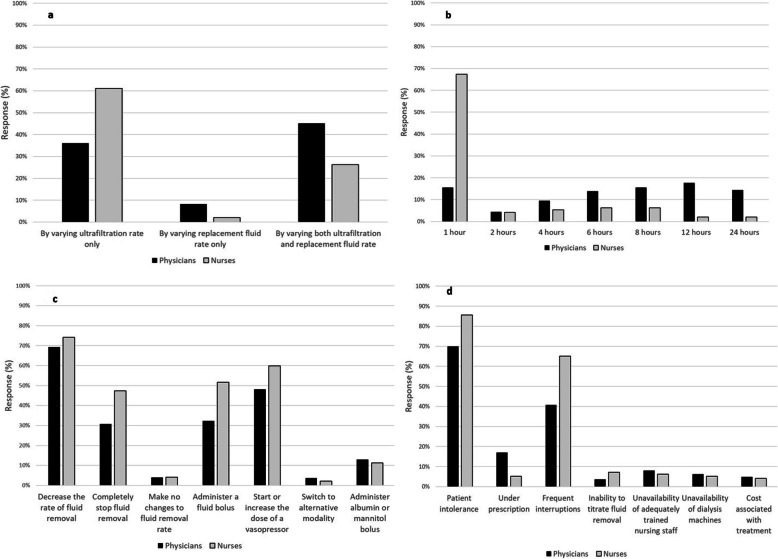
Comparison of ultrafiltration practice between physicians and nurses. **a** Methods to achieve ultrafiltration during continuous renal replacement therapy. Nurses were more likely to achieve ultrafiltration by varying ultrafiltration rate, while physicians were more likely to vary both ultrafiltration and replacement fluid rates (*p* < 0.001). **b** Frequency of net fluid balance assessment during continuous renal replacement therapy. Nurses were more likely to check fluid balance every hour, while physicians were more likely to check fluid balance every 4–24 h (*p* < 0.001). **c** Interventions to performed for hemodynamic instability during net ultrafiltration. Compared with physicians, nurses were more likely to stop fluid removal, administer a fluid bolus, and start or increase the dose of vasopressors (*p* < 0.05 for all responses). **d** Perceptions related to barriers for successful implementation of net ultrafiltration. Compared with physicians, nurses were more likely to cite barriers such as patient intolerance (*p* < 0.05), frequent interruptions (*p* < 0.001), whereas physicians were more likely to cite under prescription (*p* < 0.05)

To achieve target UF^NET^, 41.3% of participants reported that they changed only the ultrafiltration rate and 41.3% stated that they altered both the ultrafiltration and replacement fluid rates. The majority of nurses (61.6%) changed only the ultrafiltration rate (Fig. 4a). One-fourth (26.1%) of practitioners stated that they evaluated the net fluid balance routinely every 1 h. Fewer physicians evaluated the net fluid balance hourly compared to nurses (15.5% vs 67.4%; *p* < 0.001). Almost half of the physicians reported that they evaluated the net fluid balance only every 6 to 24 h (Fig. 4b).

### Hemodynamic instability and management

New hemodynamic instability, defined as new onset or worsening of tachycardia, hypotension, or a need to start or increase the dose of vasopressors, was reported as occurring in 20.0% of all patients (IQR, 10.0–30.0%). When hemodynamic instability occurred, two-thirds of practitioners (70.1%) reported that they decreased the rate of fluid removal, and half (51.3%) started a new vasopressor or increased the dose. Compared with doctors, nurses were more likely to report the following interventions: termination of fluid removal (*p* = 0.002); administration of a fluid bolus (*p* < 0.001); and initiation of vasopressor or increase of dose (*p* = 0.04) (Fig. 4c).

### Perceived barriers to successful fluid removal

Patient intolerance, as defined by practitioners, was the most common barrier to ultrafiltration (72.6%) followed by frequent treatment interruptions (45.3%) and under-prescription of UF^NET^ (15.2%). Compared with doctors, nurses were more likely to report patient intolerance (85.6% vs 69.9%; *p* = 0.002) and interruptions of treatment (65.0% vs 40.7%; *p* < 0.001), whereas physicians were more likely to report under-prescription as a barrier (17.0 vs 5.2%; *p* = 0.003) (Fig. 4d). Practitioners with more than 15 years of experience perceived under-prescription (17.6% vs 11.0%; *p* = 0.04), but fewer interruptions (40.5% vs 51.8%; *p* = 0.01) as barriers compared to those with less experience. A large proportion of practitioners working in government hospitals reported frequent treatment interruptions (61.0%), while practitioners from other types of hospital reported unavailability of dialysis machines (14.8%) as a barrier to UF^NET^.

### Attitudes related to timing, use of protocol, and enrolling patients in a

#### Clinical trial of protocol-based UF_NET_

Most respondents (90.7%) felt that early UF^NET^ would be beneficial, and that protocol-based fluid removal (77.1%) would be useful. About 80% confirmed their willingness to enroll patients into a clinical trial comparing protocol based UF^NET^ versus usual care, with physicians more willing to enroll than nurses.

#### Thematic analysis of comments

There were 133 (34.6%) and 23 (23.7%) comments from 384 doctors and 97 nurses, respectively. Thematic analysis revealed five common themes broadly categorized into equipment, organizational-, clinician-, patient-, and RRT-related factors. Of these, the comments related to functional hemodynamic monitoring (11.5%) and assessment of patients’ comorbidities (9.0%) were predominant themes (Supplementary data: Table S5 and S6).

## Discussion

In this survey of doctors and nurses working in hospitals in Europe, the respondents were diverse with respect to countries, profession, years of practice, and types of hospital. Most had more than 15 years of experience and practiced in university-based hospitals. Almost all doctors were intensivists. There was significant variation in use of diuretics, criteria for initiation of ultrafiltration, UF^NET^ prescription, assessment of prescribed-to-delivered dose, RRT modality, monitoring of net fluid balance, management of hemodynamic instability, and perceived barriers to UF. Most respondents agreed that early fluid removal might be useful, and that protocol-guided fluid removal might be beneficial. More doctors than nurses were keen to enroll patients in a clinical trial comparing a protocol-based ultrafiltration versus usual care.

The maximal dose of loop diuretics before determining diuretic resistance varied significantly. This may reflect lack of consensus in the literature. A recent survey also confirmed a wide de-resuscitation practice among critical care physicians [17]. The term ‘diuretic resistance’ is typically used in heart failure but its definition is less clear in AKI where higher doses of furosemide may be required to achieve diuresis due to reduced kidney function [18]. Consequently, the maximum dose of loop diuretics safe in AKI before considering extracorporeal fluid removal is unknown.

About half of doctors considered oliguria/anuria as a criterion for mechanical fluid removal, followed by complications of fluid overload e.g. pulmonary edema, hypoxemia. Most practitioners believed that earlier initiation might yield better outcomes. Whether this has changed following the publication of three recent randomized controlled trials and an individual-patient meta-analysis which failed to show a difference in mortality between early (within 6–12 h after AKI stage 3) versus delayed (up to 72 h after oliguria onset) initiation of RRT is unknown [12, 13, 19, 20]. Some earlier studies, including a single-center randomized controlled study with predominant surgical patients of whom 75% had pulmonary edema before randomization showed that early initiation of RRT had a survival benefit [11]. However, the largest study to date, STARRT-AKI trial, showed no difference in 90-day mortality between the accelerated strategy and standard initiation groups, despite differences in fluid balance at RRT initiation (2.7 vs 5.9 L) [20].

In Europe, CRRT is the predominant modality [15] although CRRT was used less commonly in certain types of hospitals, e.g. private hospitals due to unknown external factors e.g. availability of machines.

When prescribing UF^NET^, most respondents reported using hemodynamic parameters to guide their decision. Another international survey also showed that most physicians applied clinical examination and bedside assessment including body weight and cumulative fluid balance to determine fluid status. More advanced techniques such as echocardiography, ultrasonography, and cardiac output monitoring were infrequently used [21]. A combination of dynamic hemodynamic parameters and intravascular fluid assessment is recommended for evaluation of fluid responsiveness [22]. Nevertheless, few studies have incorporated these methods into guiding ultrafiltration and monitoring, for example, passive leg raising, trans-pulmonary blood dilution, or blood volume monitoring during RRT [23–25].

The setting of the ultrafiltration rate remains one of the most controversial issues in RRT-dependent fluid management. Our survey confirmed a vast range of initial and maximal UF targets in hemodynamically stable and unstable patients. Doctors were more likely to report under-prescribing as a barrier to achieving UF^NET^ targets compared to nurses, especially those with > 15 years of clinical experience. Previous research showed that in patients with fluid overload prior to initiation of RRT, an UF^NET^ intensity > 25 mL/kg/day (e.g. ~ 73 mL/hr. in a 70-kg patient) was associated with lower 1-year mortality [26]. In contrast, a secondary analysis of the Randomized Evaluation of Normal versus Augmented Level (RENAL) trial reported that a UF^NET^ rate > 1.75 mL/kg/h (e.g. ~ 123 mL/hr. in a 70-kg patient) was associated with a higher risk of 90-day mortality [27]. Similarly, a recent study supported that a UF^NET^ rate > 1.75 mL/kg/hr. in the first 48 h was associated with increased mortality, lower potassium, higher hypophosphatemia, a longer duration of CRRT, and longer stay in ICU [28]. Observational studies in chronic hemodialysis patients showed similar results: high rates of fluid removal were associated with increased cardiovascular mortality, possibly due to impaired plasma refilling rate and myocardial stunning [29, 30].

Frequent reassessment of fluid status and hemodynamics is essential to tailor the therapy according to the needs of the patient. Fluid removal targets should be set to promote fluid removal, to avoid unnecessary fluid administration, and to maintain end-organ perfusion. A recent analysis of 820 patients on CRRT concluded that a decrease in cumulative fluid balance during CRRT was independently associated with higher chance of survival. Importantly, the number of days without a prescribed fluid balance target was an independent risk factor for mortality [31]. A study in an Australian ICU found that the fluid targets were not met on 26% of treatment days [32]. Our survey identified that a significant proportion of respondents did not routinely assess prescribed-to-delivered net balance. Furthermore, hourly monitoring of net fluid balance during RRT was reported by only 26% of practitioners, with nurses performing the task more frequently than physicians. Physicians were more likely to manipulate UF^NET^ by varying both replacement fluid and ultrafiltration rate, while nurses were more likely to vary the ultrafiltration rate alone. Each technique has its own advantages and disadvantages, but ideally, each center should agree on one method to simplify communication and streamline clinical practice [14].

Decreasing or stopping UF^NET^ removal was a common strategy for managing patients with hemodynamic instability, and patient intolerance remained the highest concern for practitioners. Although excessive ultrafiltration is one of the main factors, there are other mechanisms that contribute to RRT-related hypotension, in particular decreased peripheral vascular resistance, impaired cardiac function, or both. Therefore, decreasing or stopping ultrafiltration might not always be appropriate especially in the context of fluid overload [33]. There is limited evidence to recommend a particular intervention to mitigating hemodynamic instability during RRT. A recent systematic review indicated that higher dialysate sodium, adjustment of UF rate, and lower temperature might be beneficial although the data were derived from small, low-quality studies, and mainly in IHD [34]. Frequent interruptions of treatment was another important reason for not achieving target UF^NET^. The importance of potentially modifiable factors including appropriate vascular access and anticoagulation should be emphasized and routinely reviewed. Lack of staff, machines, and cost were infrequently reported, which may reflect healthcare in Europe.

In Europe, UF initiation and practice is led predominantly by intensive care teams but not uniformly [35]. Nonetheless, within each center, RRT practice often varies. For example, RRT delivery is nurse-led with medical oversight in some centers, and led by medical teams in others. In many centers, the ICU nurses or nurse practitioners are authorized to administer fluid boluses, reduce or stop UF, or start vasopressor in response to hemodynamic instability according to approved institutional guidelines. These departmental differences may explain the varied responses to the survey questions related to UF methods, frequency of fluid balance monitoring, interventions following hemodynamic instability, and perceived barriers to UF between nurses and physicians. This report confirms the vast heterogeneity in RRT practice and delivery even within one continent. There is a clear need for future high-quality studies to determine whether different UF practices have effects on outcomes and to inform consensus recommendations and standardization. This issue is particularly important in the context of the current health crisis where there is an increased need for rapid scale-up of ICU capacity and personnel, including upskilling of non-ICU staff [36].

There are some limitations in this study that we would like to acknowledge. First, the respondents were predominantly intensivists. The view of nephrologists may be under-represented. Nevertheless, this reflects clinical practice in ICUs in Europe, where UF is most commonly prescribed by intensivists. The role of nephrologists in ICUs in Europe varies. In addition to providing RRT in some centers, nephrologists often have an advisory roles, for instance in cases where there is diagnostic dilemma, and take over the care of patients who may need ongoing kidney support after ICU discharge. Second, we are unable to determine the exact response rate to this survey. Six hundred seventy-nine people started the survey, but we are unsure of the denominator as the survey was emailed to members of 3 different societies, and we do not know how many people opened their email. However, this is the first and largest survey on ultrafiltration practice of doctors and nurses working in Europe to date. Third, selection bias may have been generated from those who completed the survey; information bias could also be present by each practitioner’s subjective response. Fourth, the duration of IHD and PIRRT were not included in the questionnaire, therefore we could not estimate the UF^NET^ per hour for this modality. Fifth, the responses may be skewed due to the disparate numbers of participants from some countries. About one-third of the responders are from the UK which might be a reflection of the membership of the ESICM and the fact that the main investigator (MO) was from the UK. The survey is inclusive of variably experienced physicians and nurses from different types of hospital in 31 countries. Similar to the responses from practitioners working in hospitals outside Europe, our findings confirmed that there was wide variation in UF practice across Europe [15]. Significant variations in practice accentuate the lack of evidence, which raises several important questions and serves as a basis for future research and quality improvement projects in this area. Finally, protocolized de-resuscitation by diuretics has previously been shown to improve outcomes and achieve negative balance compared to usual care but more work is necessary [37]. It is encouraging to see that the majority of respondents supported future research to identify optimal mechanical fluid management.

## Conclusion

In conclusion, there is a significant variation in fluid management, including monitoring and management of complications among doctors and nurses working in hospitals in Europe. Further studies are urgently needed to answer the unknown questions; when, how, and which tools to use to achieve UF^NET^ targets.

## Supplementary Information

**Additional file 1: Supplementary Content 1.** Survey instrument. **Supplementary Figure S1.** Proportions of practitioners and maximum doses of loop diuretics (furosemide equivalent) prescribed per day. **Supplementary Table S1.** Comparison by years of clinical practice. **Supplementary Table S2.** Comparison by types of hospitals. **Supplementary Figure S2**. Modalities of RRT use in each type of hospital. **Supplementary Table S3.** Comparison by seven top respondent countries. **Supplementary Table S4.** Net Ultrafiltration Rates by Country. **Supplementary Table S5.** Thematic Analysis of Comments by Practitioner Type. **Supplementary Table S6.** Examples of Comments Amenable to Research and Quality Improvement Interventions

## Data Availability

The datasets used and/or analyzed during the current study are available from the corresponding author on reasonable request.

## Abbreviations

AKI: Acute kidney injury
ADQI: Acute Dialysis Quality Initiative
BACCN: British Association of Critical Care Nurses
CRRT: Continuous renal replacement therapy
ESICM: European Society of Intensive Care Medicine
ICU: Intensive care unit
IQR: Interquartile range
IHD: Intermittent hemodialysis
PIRRT: Prolonged intermittent renal replacement therapy
RRT: Renal replacement therapy
SCUF: Slow continuous ultrafiltration
UF: Ultrafiltration
UFNET: Net ultrafiltration
UK: The United Kingdom
