# Supplementary material for: Net ultrafiltration prescription survey in Europe
Source: BMC Nephrol. 2020 Dec 1;21:522. doi: 10.1186/s12882-020-02184-y (PMC7706211; doi:10.1186/s12882-020-02184-y)
Supplement: Supplementary file 1 — Additional file 1: Supplementary Content 1. Survey instrument. Supplementary Figure S1. Proportions of practitioners and maximum doses of loop diuretics (furosemide equivalent) prescribed per day. Supplementary Table S1. Comparison by years of clinical practice. Supplementary Table S2. Comparison by types of hospitals. Supplementary Figure S2. Modalities of RRT use in each type of hospital. Supplementary Table S3. Comparison by seven top respondent countries. Supplementary Table S4. Net Ultrafiltration Rates by Country. Supplementary Table S5. Thematic Analysis of Comments by Practitioner Type. Supplementary Table S6. Examples of Comments Amenable to Research and Quality Improvement Interventions [file 12882_2020_2184_MOESM1_ESM.docx]

# Additional File

# Supplementary Content 1: Survey Instrument


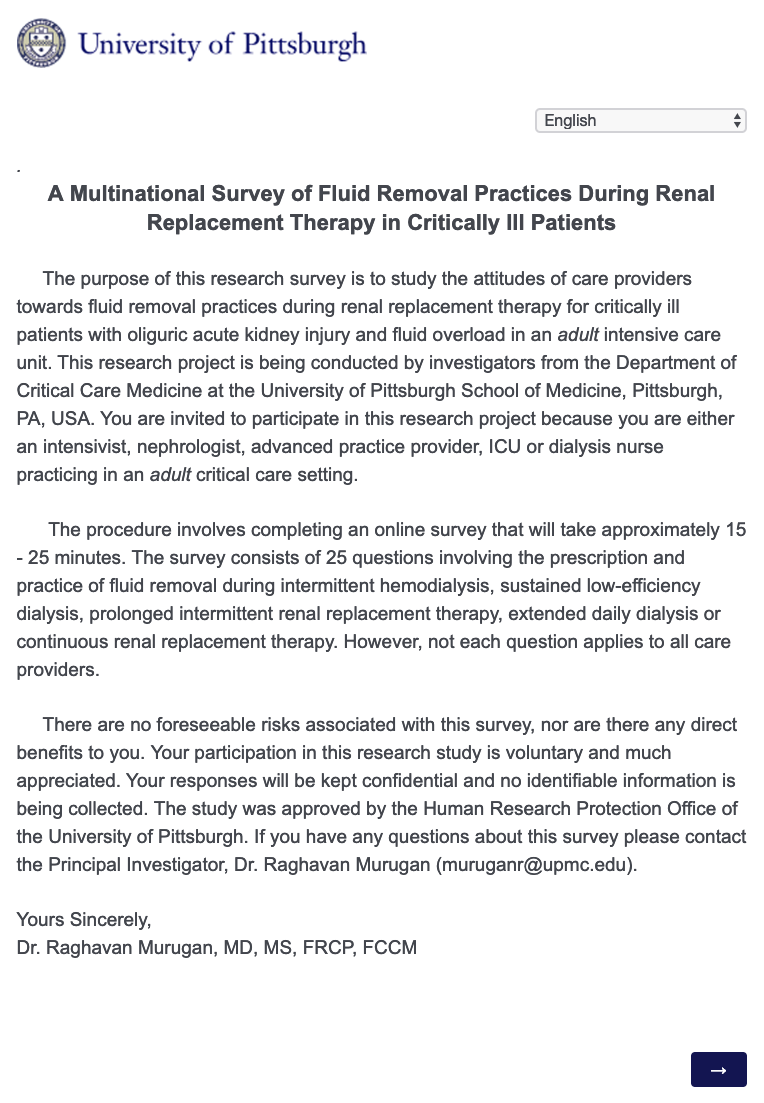


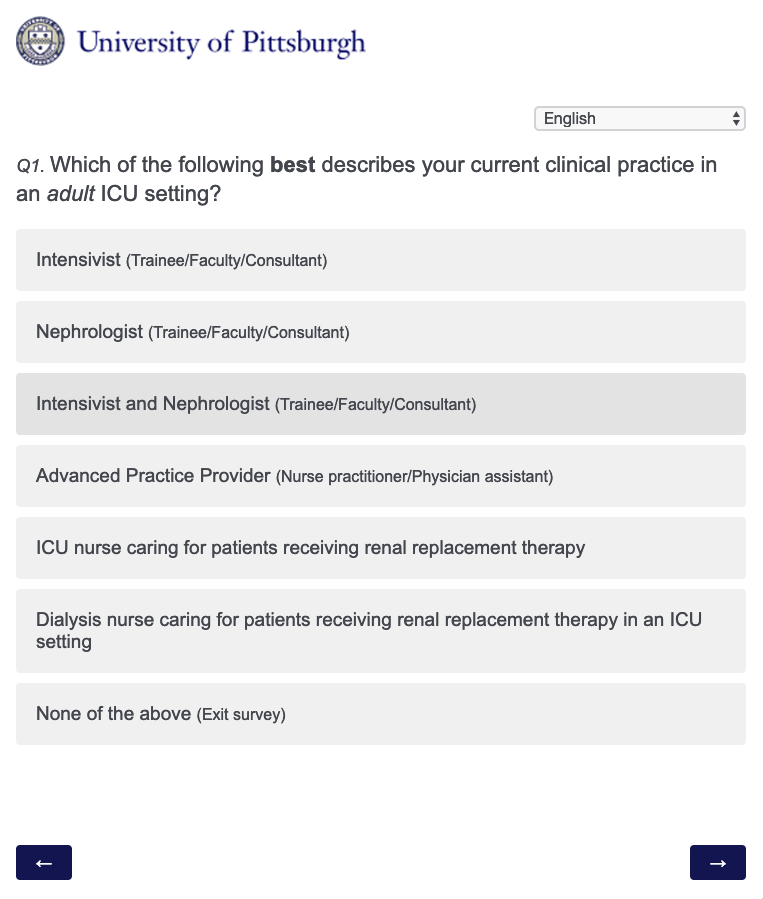


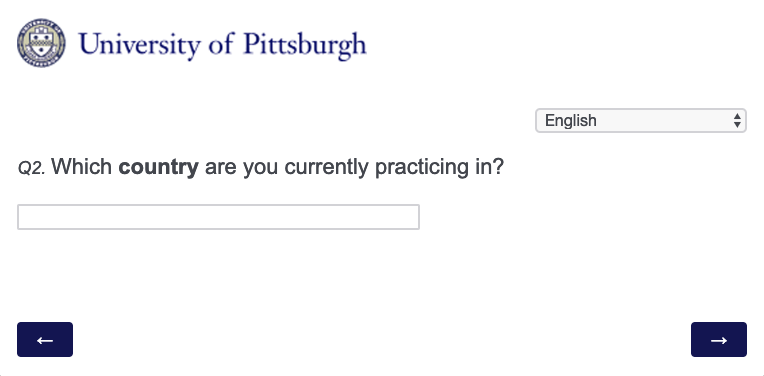


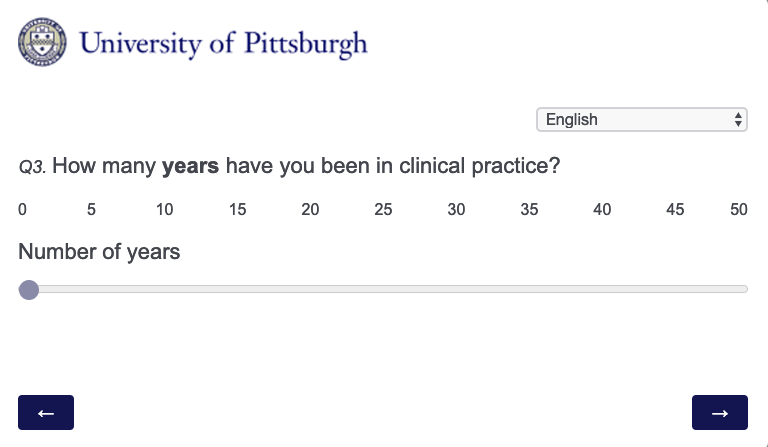


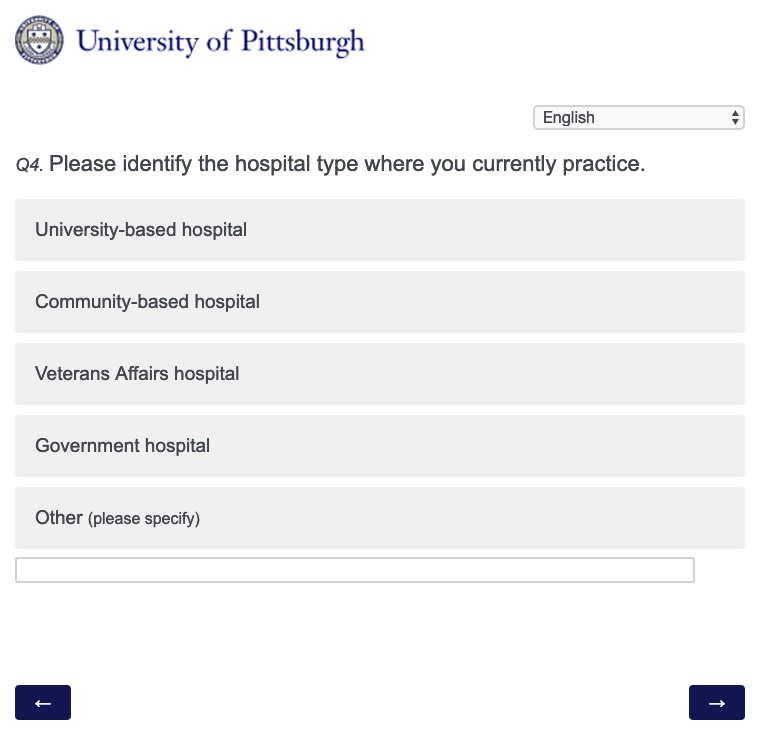


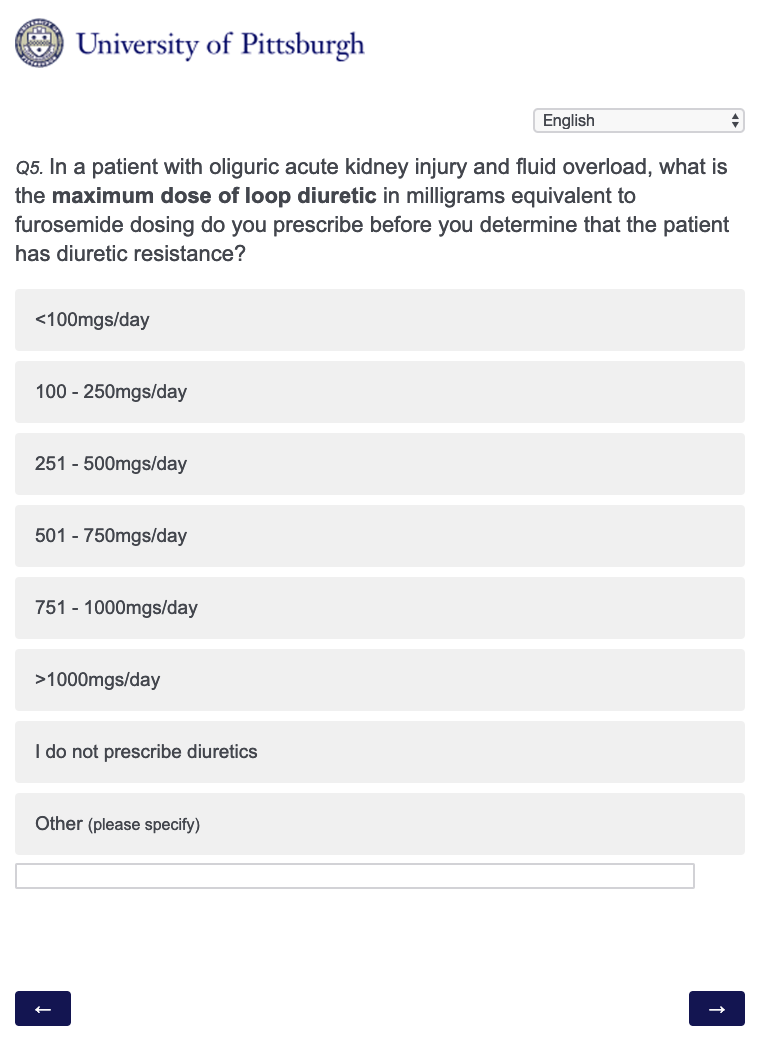


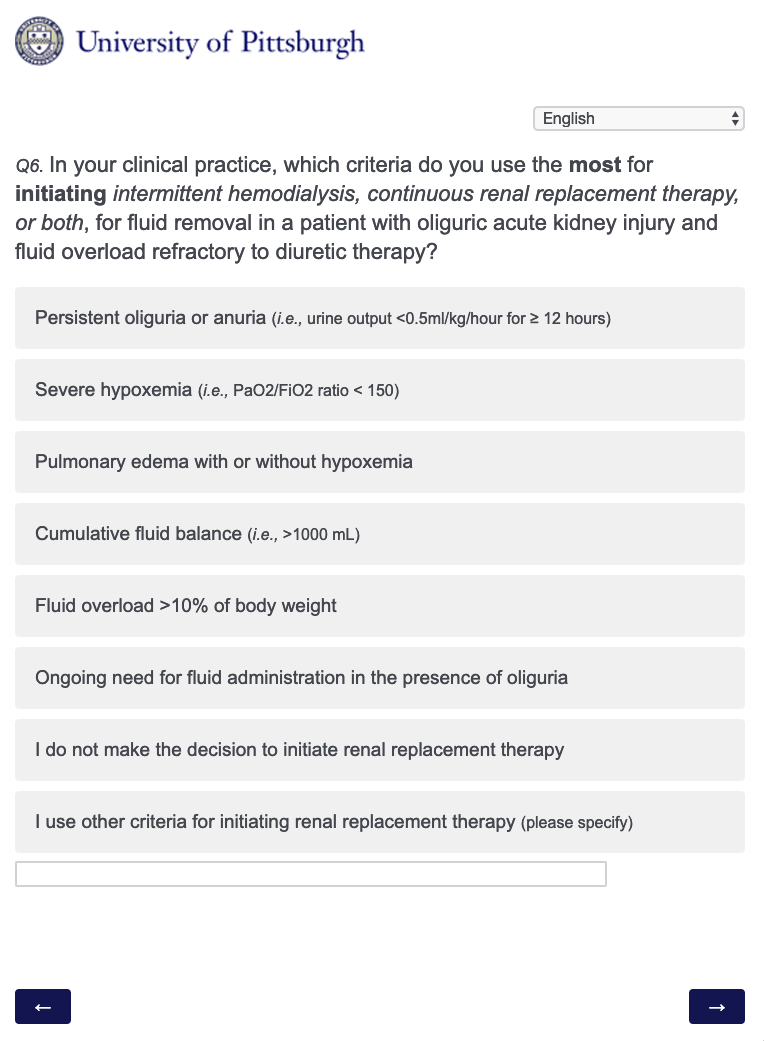

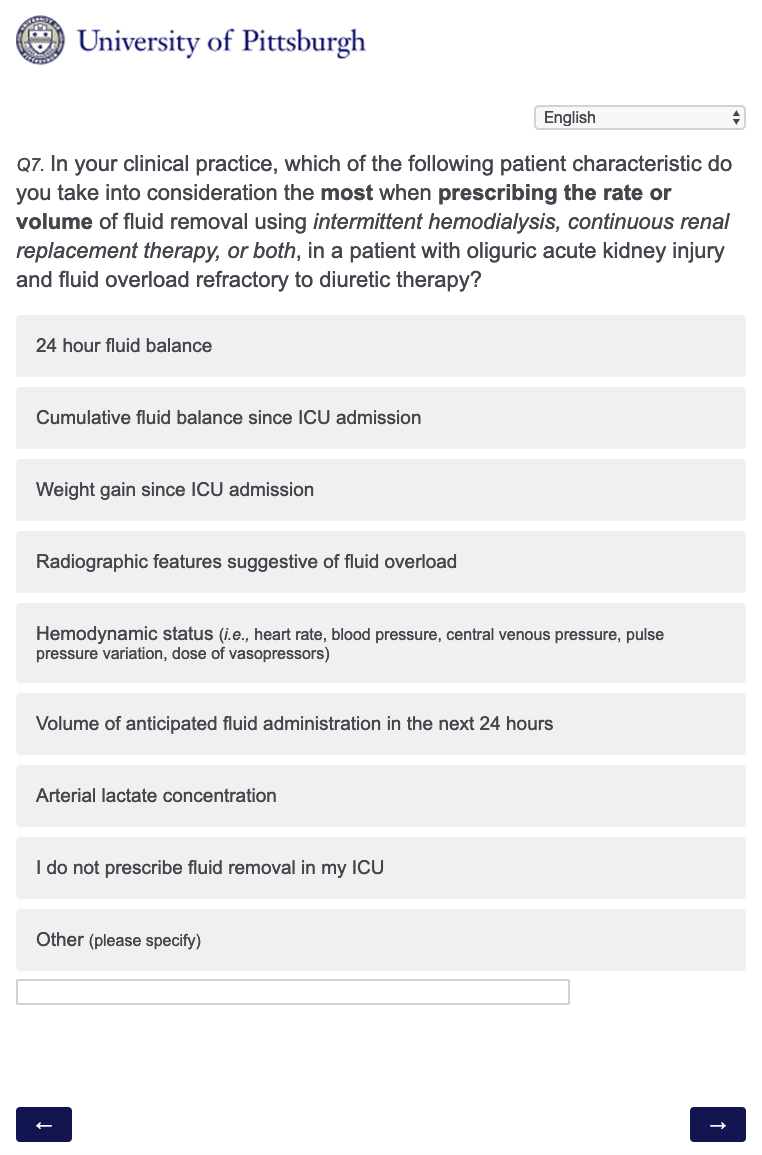

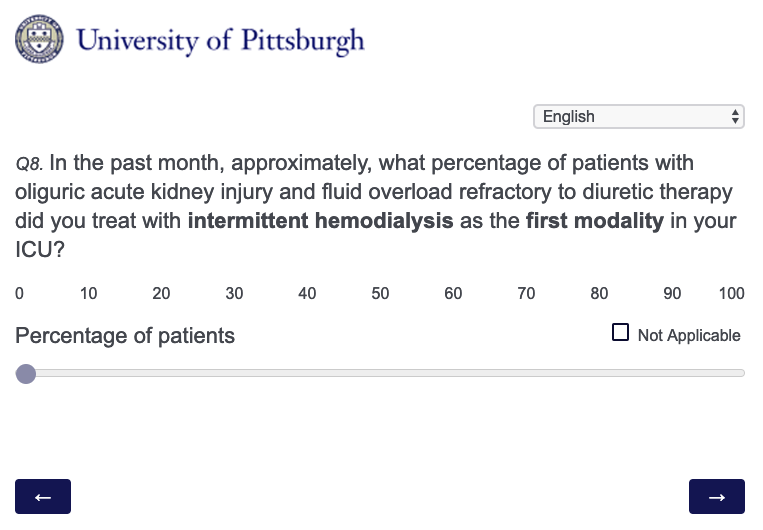

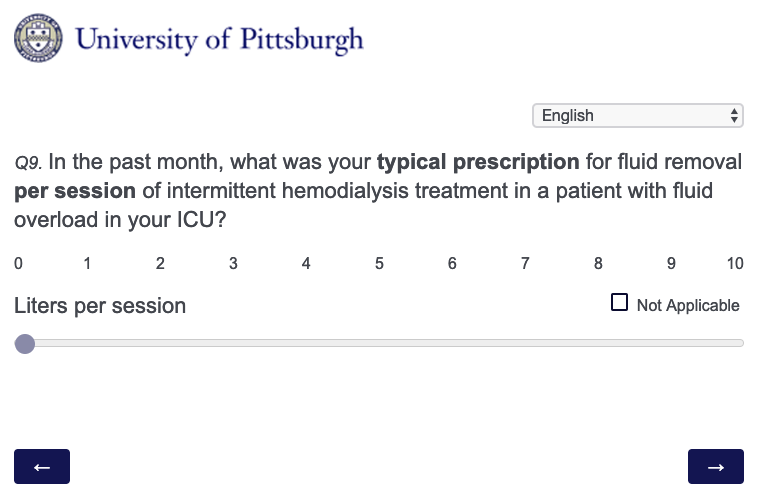

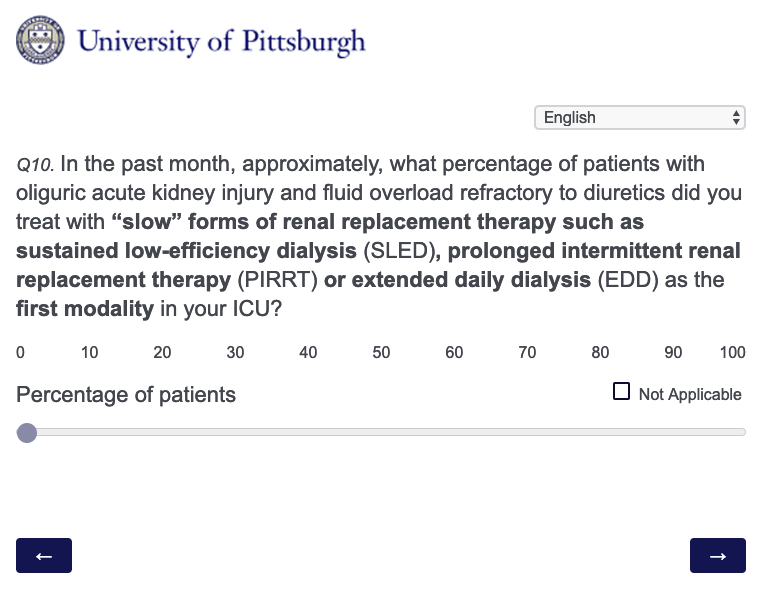

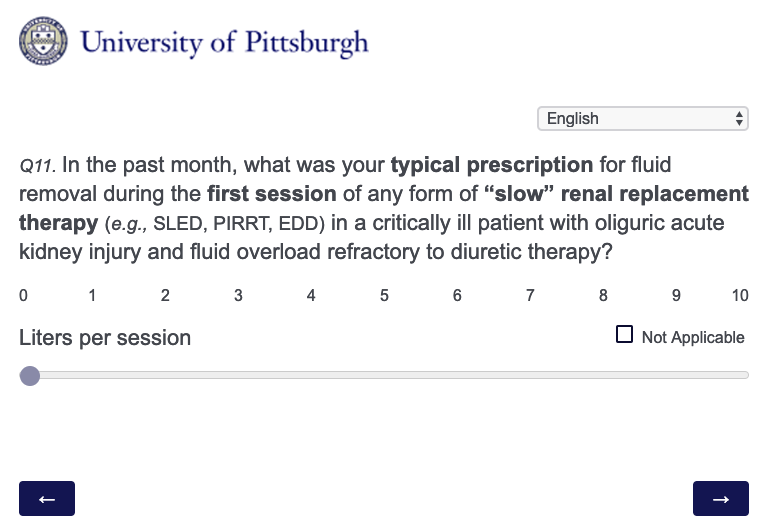

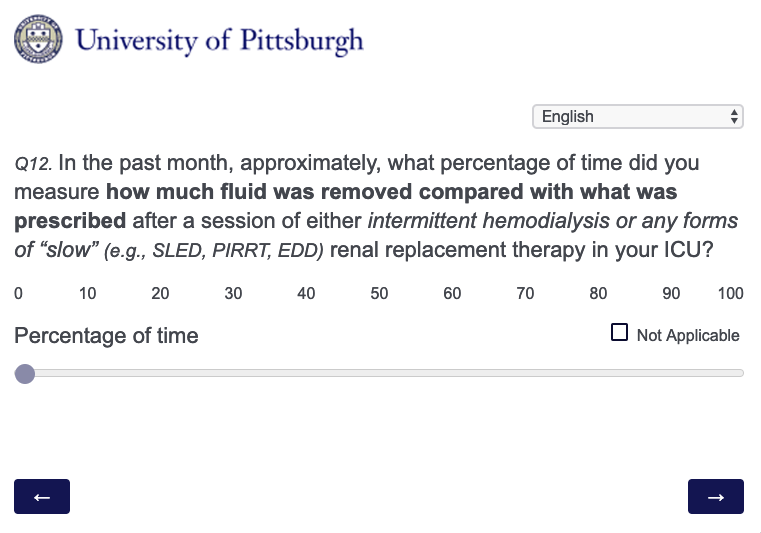

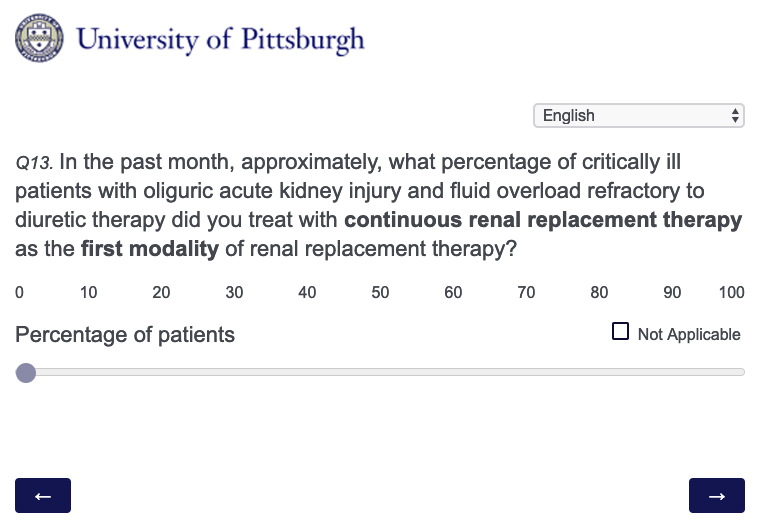

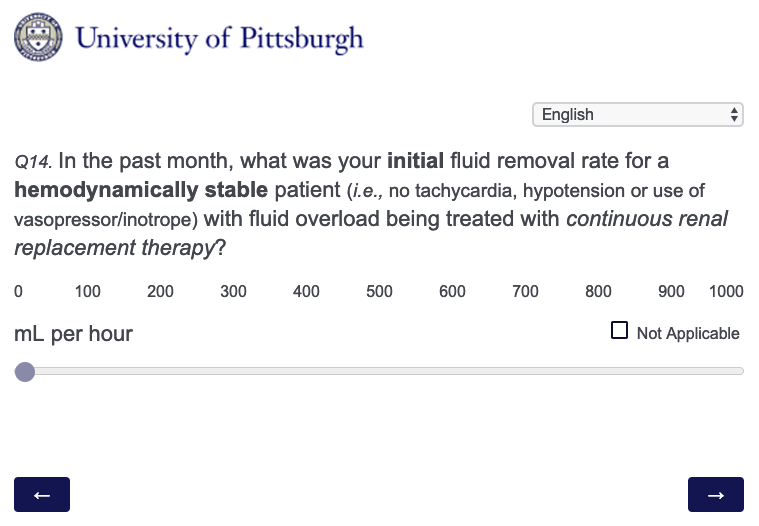

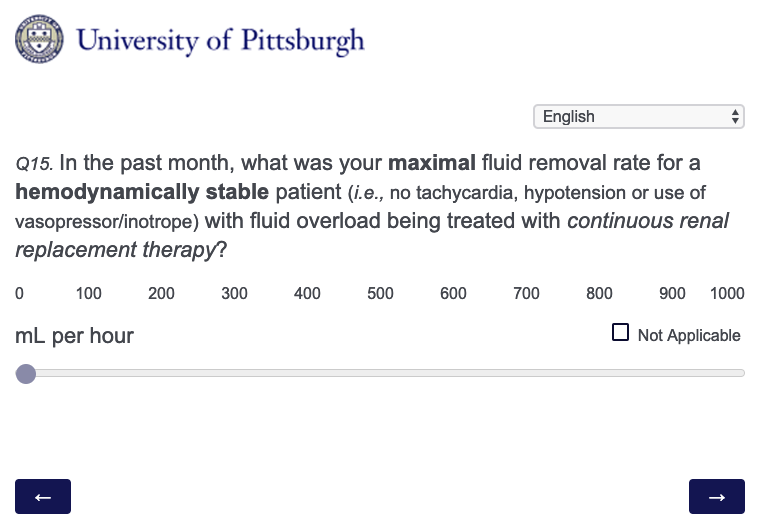

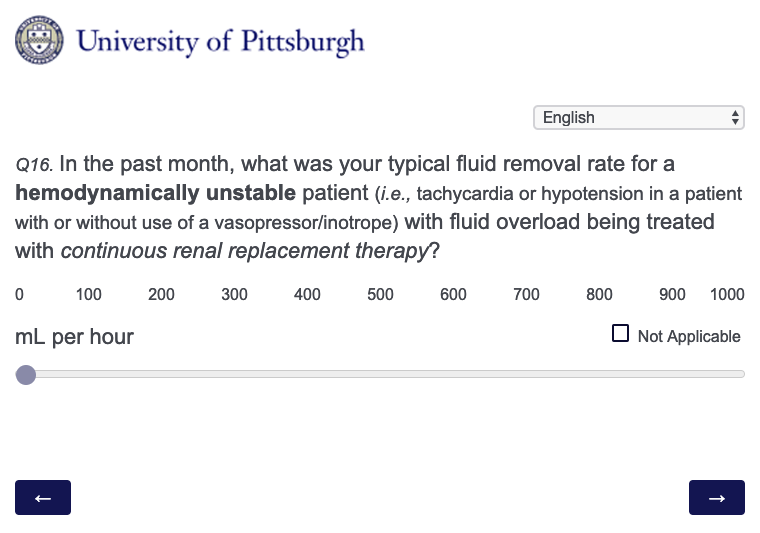

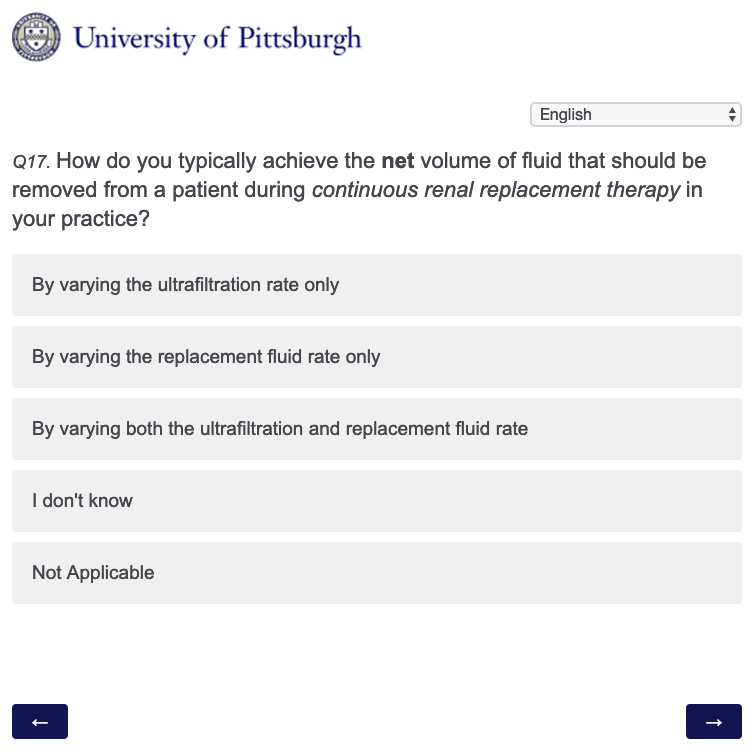

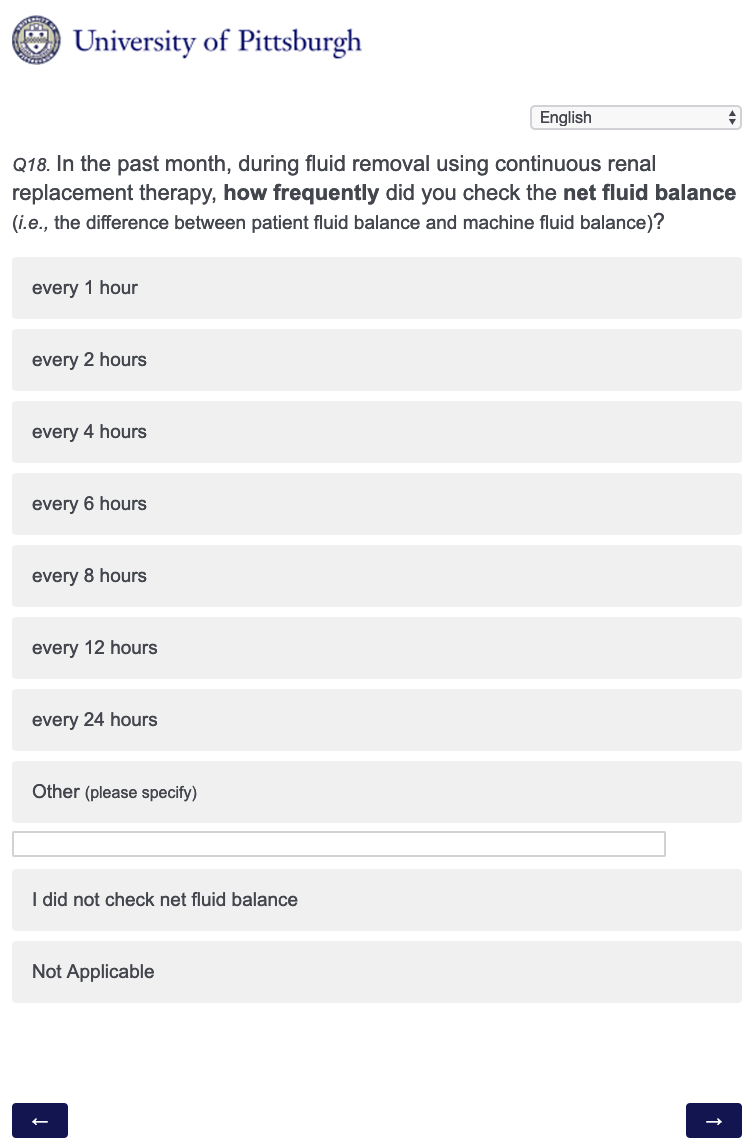

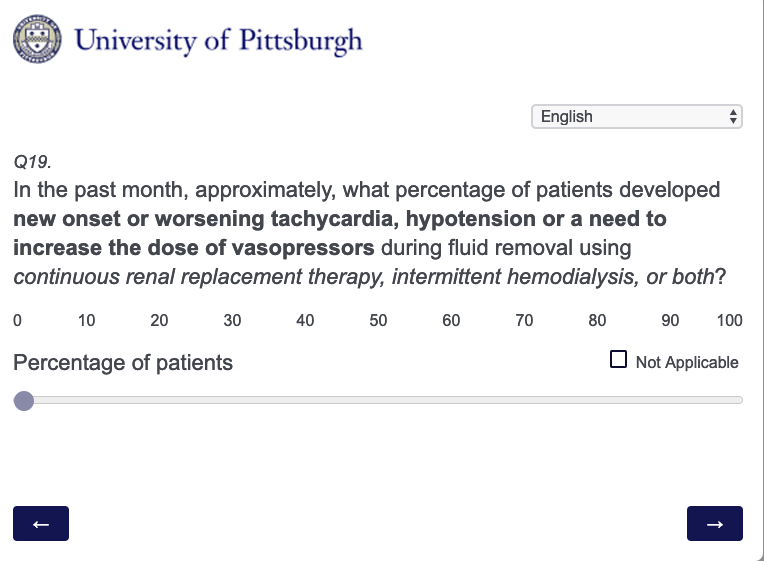

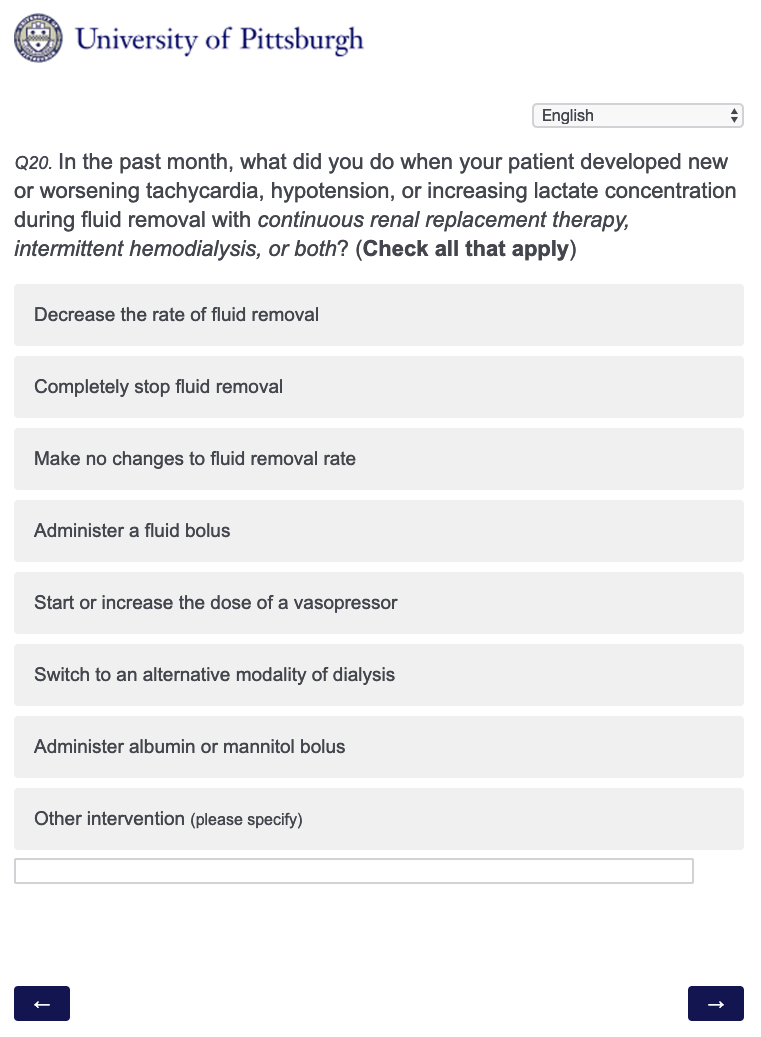

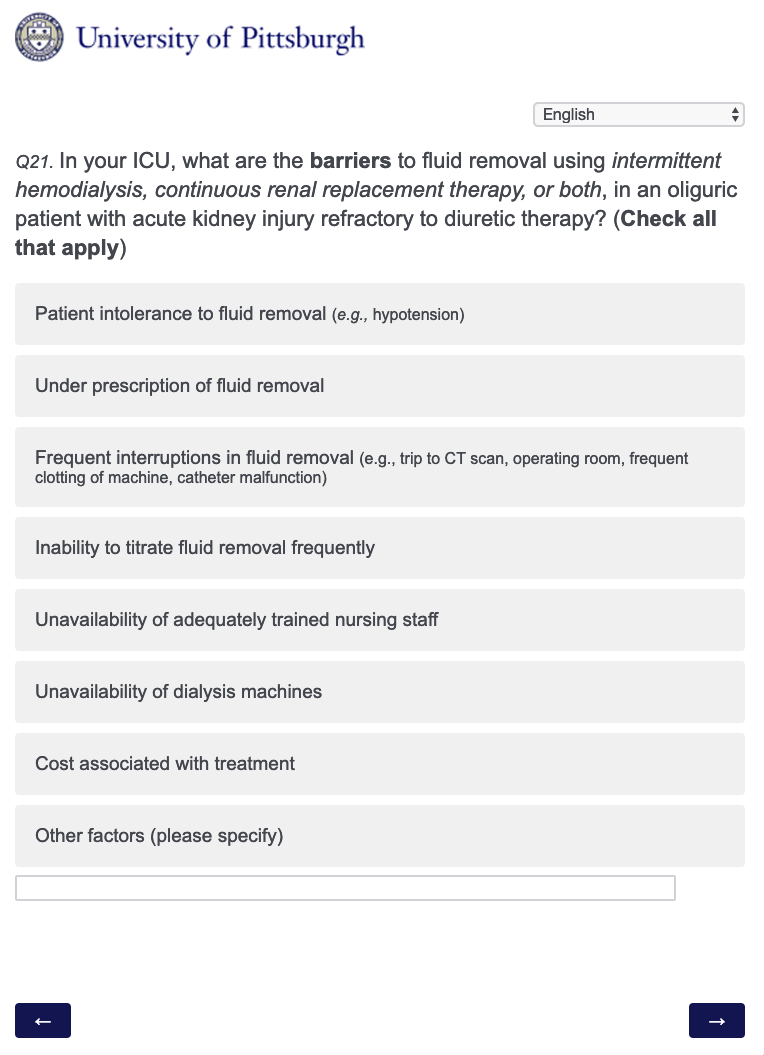

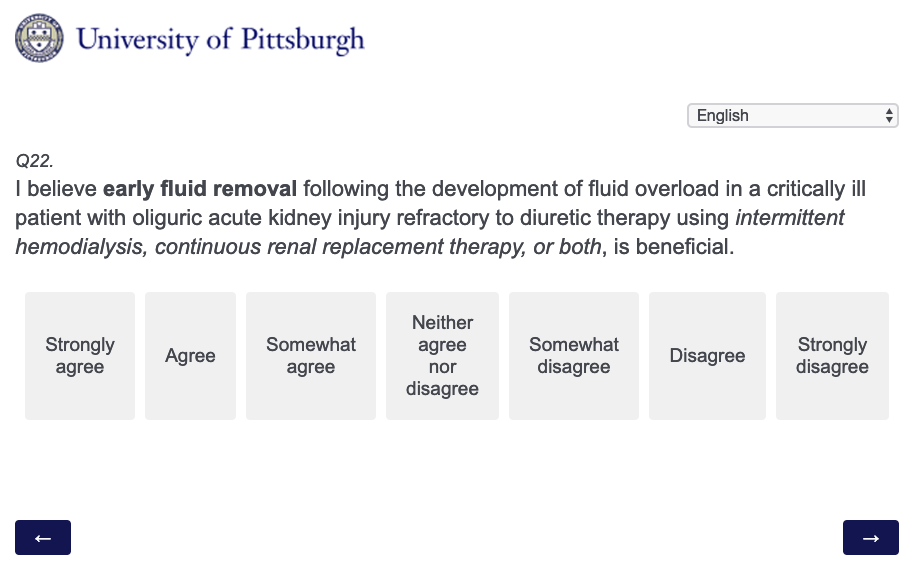

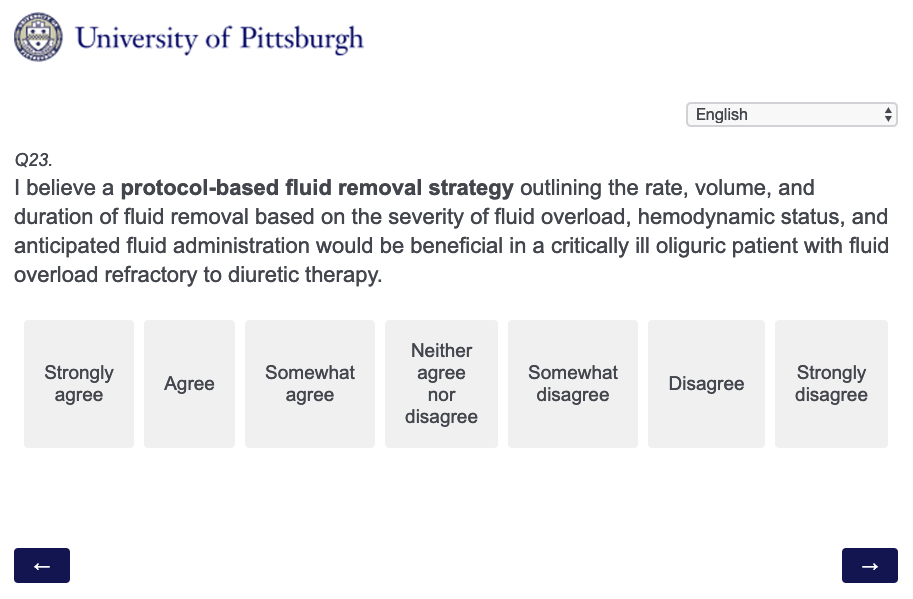

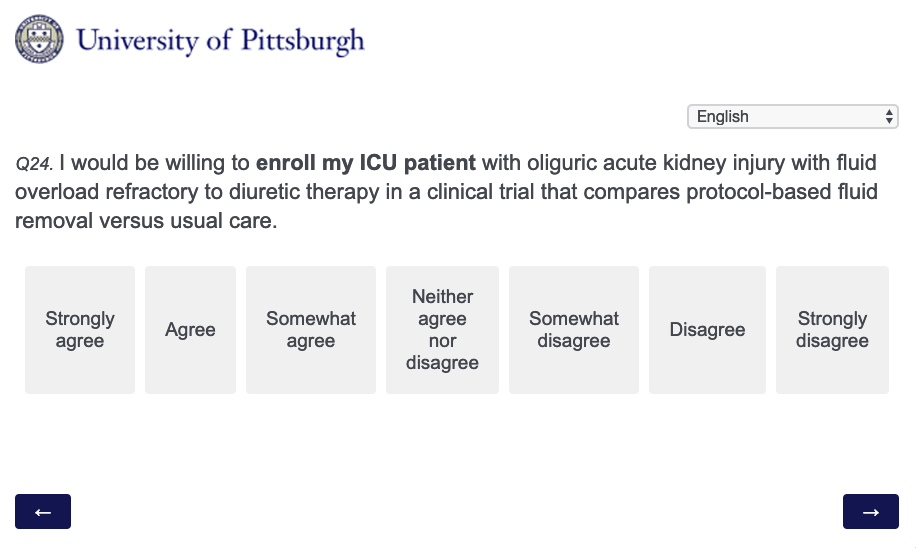

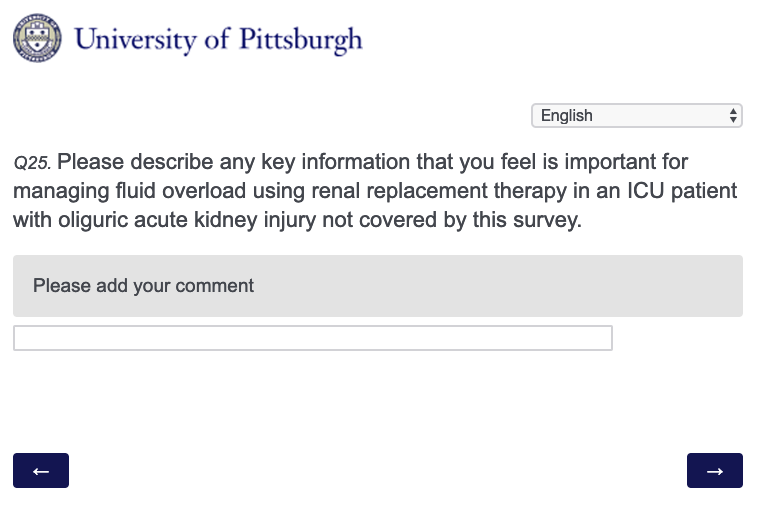


**Supplementary Figure S1** Proportions of practitioners and maximum doses of loop diuretics (furosemide equivalent) prescribed per day

**
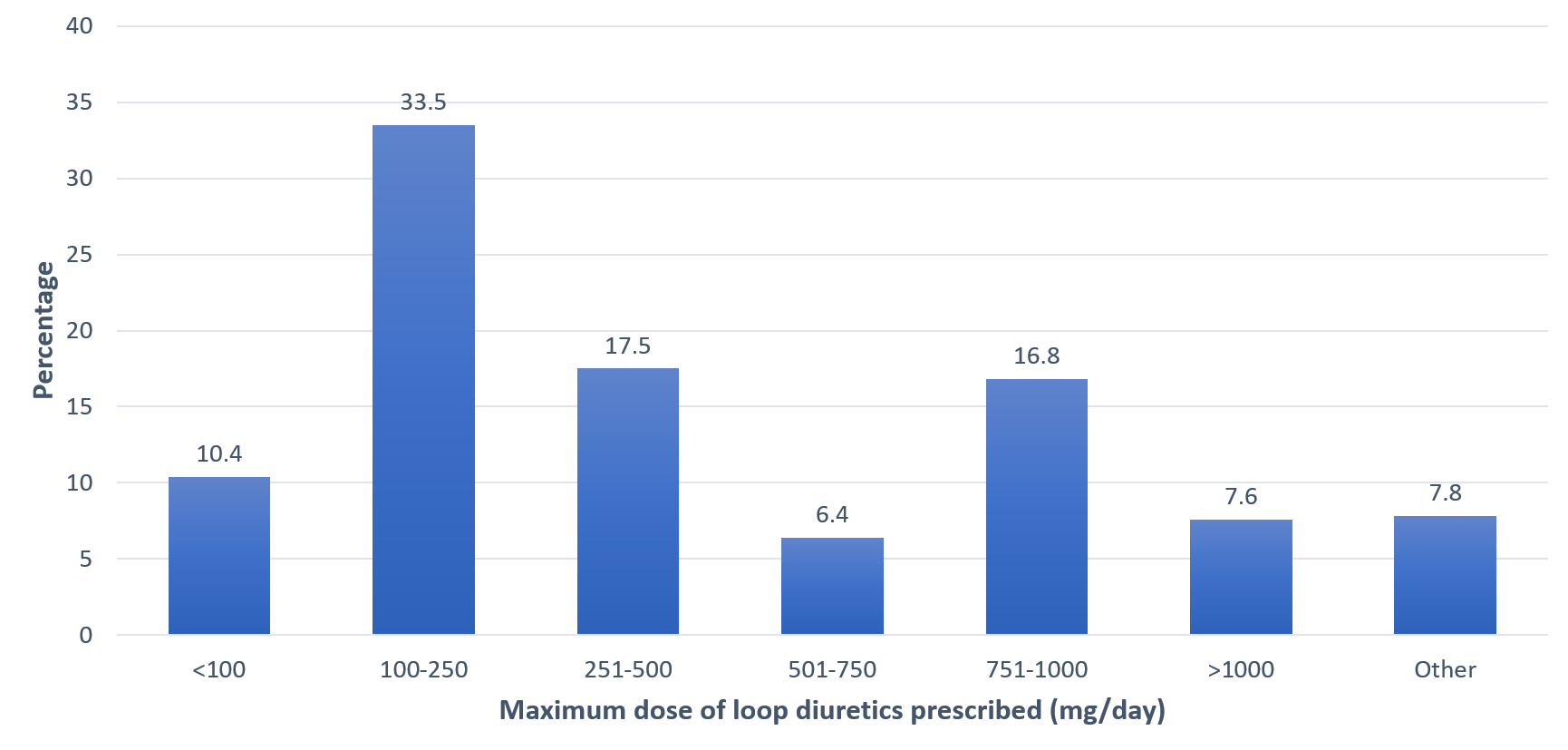
**

*Others represent usage of other doses e.g. 1-1.5 mg/kg or the practitioners do not prescribe diuretics

**Supplementary Table S1** Comparison by years of clinical practice

| **Characteristic** | | **No. (%)** | |  |
| --- | --- | --- | --- | --- |
|  |  | **< 15 years (n=218)** | **≥ 15 years (n=267)** | **P Value** |
| Country | |  |  |  |
|  | United Kingdom (n=181) | 89 (40.8) | 92 (34.5) | 0.17 |
|  | Italy (n=78) | 37 (17.0) | 41 (15.4) |  |
|  | Spain (n=31) | 15 (6.9) | 16 (6.0) |  |
|  | Greece (n=24) | 7 (3.2) | 17 (6.4) |  |
|  | France (n=22) | 6 (2.8) | 16 (6.0) |  |
|  | Portugal (n=21) | 12 (5.5) | 9 (3.4) |  |
|  | Germany (n=17) | 9 (4.1) | 8 (3.0) |  |
|  | Others (n=111) | 43 (19.7) | 68 (25.5) |  |
|  |  |  |  |  |
| Occupation | |  |  |  |
| Advanced practice practitioners | | 5 (2.3) | 2 (0.8) | **0.001** |
| Dialysis nurses | | 0 | 1 (0.4) |  |
| ICU nurses | | **58 (26.6)** | 31 (11.6) |  |
| Intensivists | | 144 (66.1) | **221 (82.8)** |  |
| Intensivist and Nephrologist | | 9 (4.1) | 10 (3.8) |  |
| Nephrologists | | 2 (0.9) | 2 (0.8) |  |
|  |  |  |  |  |
| Hospital Type | |  |  |  |
|  | University-based | 154 (70.6) | 152 (56.9) | **<0.001** |
|  | Community-based | 33 (15.1) | 77 (28.8) |  |
|  | Government | 24 (11.0) | 17 (6.4) |  |
|  | Other | 7 (3.2) | 21 (7.9) |  |
|  |  |  |  |  |
| Maximum dose of loop diuretic prescribed (furosemide equivalent)^a^, mgs/day (n=394) | |  |  |  |
|  | <100 | 14 (8.8) | 27 (11.5) | 0.051 |
|  | 100 – 250 | 60 (37.5) | 72 (30.8) |  |
|  | 251 – 500 | 37 (23.1) | 32 (13.7) |  |
|  | 501 – 750 | 11 (6.9) | 14 (6.0) |  |
|  | 751 – 1000 | 22 (13.8) | 44 (18.8) |  |
|  | >1000 | 9 (5.6) | 21 (9.0) |  |
|  | I do not prescribe diuretics. | 5 (3.1) | 18 (7.7) |  |
|  | Others | 2 (1.3) | 6 (2.6) |  |
|  |  |  |  |  |
| Criteria used for initiation of UF^NET a^ (n=395) | |  |  |  |
|  | Persistent oliguria/anuria (urine output <0.5mL/kg/hour for ≥12 hours) | 75 (46.9) | 105 (44.7) | 0.34 |
|  | Severe hypoxemia (PaO_2_/FiO_2_ ratio <150) | 16 (10.0) | 22 (9.4) |  |
|  | Pulmonary edema with or without hypoxemia | 31 (19.4) | 35 (14.9) |  |
|  | Cumulative fluid balance (>1000 mL) | 7 (4.4) | 12 (5.1) |  |
|  | Fluid overload >10% of body weight | 6 (3.8) | 15 (6.4) |  |
|  | Ongoing need for fluids in the presence of oliguria | 8 (5.0) | 18 (7.7) |  |
|  | I do not make the decision | 4 (2.5) | 1 (0.4) |  |
|  | I use other criteria. | 13 (8.1) | 27 (11.5) |  |
|  |  |  |  |  |
| Criteria used for prescription of UF^NET a^ (n=395) | |  |  |  |
|  | 24-hour fluid balance | 28 (17.5) | 35 (14.9) | 0.69 |
|  | Cumulative fluid balance | 34 (21.3) | 54 (23.0) |  |
|  | Weight gain | 8 (5.0) | 23 (9.8) |  |
|  | Radiographic features of fluid overload | 3 (1.9) | 4 (1.7) |  |
|  | Hemodynamic status (HR, BP, CVP, PPV, dose of vasopressors) | 76 (47.5) | 101 (43.0) |  |
|  | Volume of anticipated fluid use in the next 24 hours | 4 (2.5) | 6 (2.6) |  |
|  | Arterial lactate | 1 (0.6) | 0 |  |
|  | I do not prescribe UF. | 1 (0.6) | 2 (0.9) |  |
|  | Others | 5 (3.1) | 10 (4.3) |  |
|  |  |  |  |  |
| IHD use, median (IQR) | |  |  |  |
|  | Percent use last month | 6.0 (0-25.0) | 5.0 (0-25.0) | 0.55 |
|  | Typical prescription, mL per session | 2000 (1500-3000) | 2000 (1500-3000) | 0.70 |
|  |  |  |  |  |
| Slow forms of IHD use, median (IQR) | |  |  |  |
|  | Percent use last month | 1.0 (0-25.0) | 1.0 (0-25.0) | 0.16 |
|  | Typical prescription, mL per session | 1575 ± 1071 | 2328 ± 1512 | **0.0006** |
|  |  |  |  |  |
| Percent of assessment of prescription-to-delivered UF^NET^, median (IQR) | | 81.0 (29.0-100.0) | 75.0 (20.0-100.0) | 0.70 |
|  |  |  |  |  |
| CRRT use, median (IQR) | |  |  |  |
|  | Percent use in the last month | 82.0 (30.0-100.0) | 90.0 (33.0-100.0) | 0.39 |
|  | Initial UF^NET^ rate for hemodynamically stable patient, mL per hour | 149.5 (100-200) | 149 (100-200) | 0.52 |
|  | Maximal UF^NET^ rate for hemodynamically stable patient, mL per hour | 259 (200-350.5) | 300 (200-386) | 0.16 |
|  | UF^NET^ rate for hemodynamically unstable patient, mL per hour | 76 (49-101) | 100 (51-120) | **0.02** |
|  |  |  |  |  |
| Method used to achieve UF^NET^ using CRRT, No. (%) (n=463) | |  |  |  |
|  | By varying ultrafiltration rate only | 98 (46.7) | 93 (36.8) | 0.06 |
|  | By varying replacement fluid rate only | 11 (5.2) | 21 (8.3) |  |
|  | By varying both ultrafiltration and replacement fluid rate | 79 (37.6) | 112 (44.3) |  |
|  | I do not know. | 19 (9.1) | 17 (6.7) |  |
|  | I do not prescribe UF. | 3 (1.4) | 10 (4.0) |  |
|  |  |  |  |  |
| How frequently did you check net fluid balance during CRRT? No. (%) (n=463) | |  |  |  |
|  | 1 hour | 59 (28.1) | 62 (24.5) |  |
|  | 2 hours | 8 (3.8) | 12 (4.7) |  |
|  | 4 hours | 17 (8.1) | 23 (9.1) |  |
|  | 6 hours | 25 (11.9) | 32 (12.7) |  |
|  | 8 hours | 29 (13.8) | 34 (13.4) |  |
|  | 12 hours | 34 (16.2) | 33 (13.0 |  |
|  | 24 hours | 18 (8.6) | 37 (14.6) |  |
|  | I do not check net fluid balance. | 20 (9.5) | 20 (7.9) |  |
|  |  |  |  |  |
| Percentage of patients developing new hemodynamic instability during UF^NET^, median (IQR) | | 20.0 (10.0-30.0) | 19.0 (9.0-27.0) | **0.03** |
|  |  |  |  |  |
| Interventions performed for hemodynamic instability | |  |  |  |
|  | Decrease the rate of fluid removal | 159 (72.9) | 182 (68.2) | 0.25 |
|  | Completely stop fluid removal | 80 (36.7) | 85 (31.8) | 0.26 |
|  | Make no changes to fluid removal rate | 12 (5.5) | 7 (2.6) | 0.10 |
|  | Administer a fluid bolus | 68 (31.2) | 107 (40.1) | **0.04** |
|  | Start or increase the dose of a vasopressor | 111 (50.9) | 134 (50.2) | 0.87 |
|  | Switch to alternative modality | 10 (4.6) | 6 (2.3) | 0.15 |
|  | Administer albumin or mannitol bolus | 21 (9.6) | 40 (15.0) | 0.08 |
|  |  |  |  |  |
| Perceived barriers to UF^NET^ | |  |  |  |
|  | Patient intolerance (*e.g.,* hypotension) | 158 (72.5) | 196 (73.4) | 0.82 |
|  | Under prescription | 24 (11.0) | 47 (17.6) | **0.04** |
|  | Frequent interruptions (*e.g.,* trip to CT scan, operating room, filter clotting, catheter malfunction) | 113 (51.8) | 108 (40.5) | **0.01** |
|  | Inability to titrate fluid removal | 10 (4.6) | 11 (4.1) | 0.80 |
|  | Unavailability of adequately trained nursing staff | 21 (9.6) | 16 (6.0) | 0.13 |
|  | Unavailability of dialysis machines | 12 (5.5) | 17 (6.4) | 0.69 |
|  | Cost associated with treatment | 11 (5.1) | 12 (4.5) | 0.78 |
|  |  |  |  |  |
| I believe early fluid removal is beneficial | |  |  |  |
|  | Strongly agree | 65 (29.8) | 94 (35.2) | 0.20 |
|  | Agree | 93 (42.7) | 102 (38.2) |  |
|  | Somewhat agree | 43 (19.7) | 43 (16.1) |  |
|  | Neither agree nor disagree | 12 (5.5) | 22 (8.2) |  |
|  | Somewhat disagree | 5 (2.3) | 3 (1.1) |  |
|  | Disagree | 0 | 3 (1.1) |  |
|  | Strongly disagree | 0 | 0 |  |
|  |  |  |  |  |
| I believe a protocol-based fluid removal strategy would be beneficial | |  |  |  |
|  | Strongly agree | 55 (22.9) | 73 (27.3) | 0.059 |
|  | Agree | 68 (31.2) | 80 (30.0) |  |
|  | Somewhat agree | 53 (24.3) | 50 (18.7) |  |
|  | Neither agree nor disagree | 16 (7.3) | 36 (13.5) |  |
|  | Somewhat disagree | 11 (5.1) | 17 (6.4) |  |
|  | Disagree | 14 (6.4) | 8 (3.0) |  |
|  | Strongly disagree | 6 (2.8) | 3 (1.1) |  |
|  |  |  |  |  |
| I would enroll my patient in a clinical trial comparing protocol-based versus usual care | |  |  |  |
|  | Strongly agree | 59 (27.1) | 68 (25.6) | 0.55 |
|  | Agree | 93 (42.7) | 102 (38.4) |  |
|  | Somewhat agree | 27 (12.4) | 45 (16.9) |  |
|  | Neither agree nor disagree | 28 (12.8) | 33 (12.4) |  |
|  | Somewhat disagree | 5 (2.3) | 6 (2.3) |  |
|  | Disagree | 4 (1.8) | 11 (4.1) |  |
|  | Strongly disagree | 2 (0.9) | 1 (0.4) |  |

Abbreviations: CRRT, continuous renal replacement therapy; HR, heart rate; BP, blood pressure; CVP, central venous pressure; PPV, pulse pressure variation; IHD, intermittent haemodialysis; IQR, interquartile range; UF^net^, net ultrafiltration

^a^ Practitioners included intensivists, nephrologists, intensivists and nephrologists, and advanced practice providers. ICU and dialysis nurses were excluded from these questions.

**Supplementary Table S2** Comparison by types of hospitals

| **Characteristic** | | **No. (%)** | | | |  |
| --- | --- | --- | --- | --- | --- | --- |
|  |  | **Community (n=110)** | **Government (n=41)** | **University (n=307)** | **Others (n=27)** | **P Value** |
| Occupation | |  |  |  |  |  |
| Physicians | | 103 (93.6) | **19 (46.3)** | 248 (80.8) | 18 (66.7) | **<0.001** |
| Nurses/nurse practitioners | | 7 (6.4) | **22 (53.7)** | 59 (19.2) | 9 (33.3) |  |
|  |  |  |  |  |  |  |
| Years of practice, median (IQR) | | 20 (14-25.3) | 14 (9.9-19) | 14.8 (8-22) | 20.6 (14.9-29) | **<0.001** |
| Maximum dose of loop diuretic prescribed (furosemide equivalent) ^a^, mgs/day (n=394) | |  |  |  |  |  |
|  | <100 | 12 (11.5) | 0 | 27 (10.7) | 2 (11.1) | 0.81 |
|  | 100 – 250 | 28 (26.9) | 8 (40) | 88 (34.9) | 8 (44.4) |  |
|  | 251 – 500 | 20 (19.2) | 5 (25) | 42 (16.7) | 2 (11.1) |  |
|  | 501 – 750 | 8 (7.7) | 1 (5.0) | 15 (6.0) | 1 (5.6) |  |
|  | 751 – 1000 | 20 (19.2) | 3 (15.0) | 41 (16.3) | 2 (11.1) |  |
|  | >1000 | 10 (9.6) | 0 | 18 (7.1) | 2 (11.1) |  |
|  | Others | 6 (5.7) | 3 (15.0) | 21 (8.3) | 1 (5.6) |  |
|  |  |  |  |  |  |  |
| Criteria used for initiation of UF^NET a^ (n=395) | |  |  |  |  |  |
|  | Persistent oliguria/anuria (urine output <0.5mL/kg/hour for ≥12 hours) | 53 (51.0) | 7 (35.0) | 111 (43.9) | 9 (50) | 0.54 |
|  | Severe hypoxemia (PaO_2_/FiO_2_ ratio <150) | 11 (10.6) | 2 (10) | 24 (9.5) | 1 (5.6) |  |
|  | Pulmonary edema with or without hypoxemia | 14 (13.5) | 5 (25) | 46 (18.2) | 1 (5.6) |  |
|  | Cumulative fluid balance (>1000 mL) | 2 (1.9) | 2 (10) | 13 (5.1) | 2 (11.1) |  |
|  | Fluid overload >10% of body weight | 4 (3.9) | 2 (10) | 13 (5.1) | 2 (11.1) |  |
|  | Ongoing need for fluids in the presence of oliguria | 7 (6.7) | 2 (10) | 17 (6.7) | 0 |  |
|  | I do not make the decision/others | 13 (12.5) | 0 | 29 (11.5) | 6 (16.7) |  |
|  |  |  |  |  |  |  |
| Criteria used for prescription of UF^NET a^ (n=395) | |  |  |  |  |  |
|  | 24-hour fluid balance | 21 (20.2) | 1 (5.0) | 39 (15.4) | 2 (11.1) | 0.66 |
|  | Cumulative fluid balance | 17 (16.4) | 9 (45) | 58 (22.9) | 4 (22.2) |  |
|  | Weight gain | 10 (9.6) | 0 | 19 (7.5) | 2 (11.1) |  |
|  | Radiographic features of fluid overload | 1 (1.0) | 0 | 6 (2.4) | 0 |  |
|  | Hemodynamic status (HR, BP, CVP, PPV, dose of vasopressors) | 46 (44.2) | 8 (40) | 114 (45.1) | 9 (50.0) |  |
|  | Volume of anticipated fluid use in the next 24 hours | 3 (2.9) | 2 (10) | 5 (2.0) | 0 |  |
|  | Arterial lactate | 0 | 0 | 1 (0.4) | 0 |  |
|  | I do not prescribe UF./Others | 6 (5.8) | 0 (0.0) | 11 (4.4) | 1 (5.6) |  |
|  |  |  |  |  |  |  |
| IHD use, median (IQR) | |  |  |  |  |  |
|  | Percent use last month | 9.0 (0-29.0) | 8 (0-30.0) | 5 (0-20.0) | **15 (2-27)** | **0.04** |
|  | Typical prescription, mL per session | 2000  (2000-3000) | 2100  (1000-3000) | 2000  (1500-3000) | 2500 (2000-3000) | 0.36 |
|  |  |  |  |  |  |  |
| Slow forms of IHD use, median (IQR) | |  |  |  |  |  |
|  | Percent use last month | 1 (0-30) | 2.5 (0-21) | 0 (0-17) | 5 (0-20) | 0.61 |
|  | Typical prescription, mL per session | 2000  (1400-3000) | 500  (0-3000) | 2000 (900-2500) | 2600 (1700-5000) | **0.03** |
|  |  |  |  |  |  |  |
| Percent of assessment of prescription-to-delivered UF^NET^, median (IQR) | | 72 (29-100) | 71 (16-100) | 80 (21-100) | 71 (50-100) | 0.85 |
|  |  |  |  |  |  |  |
| CRRT use, median (IQR) | |  |  |  |  |  |
|  | Percent use in the last month | 66 (19-100) | 72 (22.5-100) | 90 (50-100) | 68 (10-100) | **0.003** |
|  | Initial UF^NET^ rate for hemodynamically stable patient, mL per hour | 107 (100-200) | 149.5 (100-200) | 151 (100-200) | 150.5 (101-200) | 0.55 |
|  | Maximal UF^NET^ rate for hemodynamically stable patient, mL per hour | 251 (200-352) | 300 (251-350.5) | 300 (203-387) | 252 (200-347) | 0.33 |
|  | UF^NET^ rate for hemodynamically unstable patient, mL per hour | 83 (50-106) | 100 (52-126) | 99 (51-108) | 65.5 (45-100) | 0.70 |
|  |  |  |  |  |  |  |
| Method used to achieve UF^NET^ using CRRT, No. (%) (n=463) | |  |  |  |  |  |
|  | By varying ultrafiltration rate only | 38 (38) | 24 (61.5) | 122 (40.8) | 7 (28.0) | 0.14 |
|  | By varying replacement fluid rate only | 7 (7) | 2 (5.1) | 21 (7.0) | 2 (8.0) |  |
|  | By varying both ultrafiltration and replacement fluid rate | 43 (43) | 12 (30.8) | 122 (40.8) | 14 (56.0) |  |
|  | I do not know. | 6 (6.0) | 0 (0.0) | 28 (9.4) | 2 (8.0) |  |
|  | I do not prescribe UF. | 6 (6.0) | 1 (2.6) | 6 (2.0) | 0 |  |
|  |  |  |  |  |  |  |
| How frequently do you check net fluid balance during CRRT? No. (%) (n=463) | |  |  |  |  |  |
|  | 1 hour | 13 (13.0) | 22 (56.4) | 79 (26.4) | 7 (28) | **0.003** |
|  | 2 hours | 8 (8.0) | 0 | 10 (3.3) | 2 (8.0) |  |
|  | 4 hours | 7 (7.0) | 4 (10.3) | 26 (8.7) | 3 (12.0) |  |
|  | 6 hours | 13 (13.0) | 2 (5.1) | 39 (13.0) | 3 (12.0) |  |
|  | 8 hours | 23 (23.0) | 3 (7.7) | 35 (11.7) | 2 (8.0) |  |
|  | 12 hours | 18 (18.0) | 0 | 44 (14.7) | 5 (20) |  |
|  | 24 hours | 12 (12.0) | 3 (7.7) | 37 (12.4) | 3 (12.0) |  |
|  | I do not check net fluid balance. | 5 (6 (6.0) | 5 (12.8) | 29 (9.7) | 0 (0.0) |  |
|  |  |  |  |  |  |  |
| Percentage of patients developing new hemodynamic instability during UF^NET^, median (IQR) | | 15 (5.5-25) | 20 (10-30) | 20 (10-30) | 20 (10-30) | 0.32 |
|  |  |  |  |  |  |  |
| Interventions performed for hemodynamic instability | |  |  |  |  |  |
|  | Decrease the rate of fluid removal | 81 (73.6) | 29 (70.7) | 213 (69.4) | 18 (66.7) | 0.83 |
|  | Completely stop fluid removal | 30 (27.3) | 14 (34.2) | 115 (37.5) | 6 (22.2) | 0.14 |
|  | Make no changes to fluid removal rate | 3 (2.7) | 4 (9.8) | 12 (3.9) | 0 | 0.16 |
|  | Administer a fluid bolus | 30 (27.3) | 16 (39.0) | 116 (37.8) | 13 (48.2) | 0.11 |
|  | Start or increase the dose of a vasopressor | 50 (45.6) | 22 (53.7) | 158 (51.5) | 15 (55.6) | 0.64 |
|  | Switch to alternative modality | 2 (1.8) | 2 (4.9) | 10 (3.3) | 2 (7.4) | 0.47 |
|  | Administer albumin or mannitol bolus | 17 (15.6) | 6 (14.6) | 36 (11.7) | 2 (7.4) | 0.61 |
|  |  |  |  |  |  |  |
| Perceived barriers to UF^NET^ | |  |  |  |  |  |
|  | Patient intolerance (*e.g.,* hypotension) | 82 (74.6) | 33 (80.5) | 224 (73.0) | 15 (55.6) | 0.14 |
|  | Under prescription | 16 (14.6) | 3 (7.3) | 46 (15.0) | 6 (22.2) | 0.39 |
|  | Frequent interruptions (*e.g.,* trip to CT scan, operating room, filter clotting, catheter malfunction) | 38 (34.6) | 25 (61.0) | 144 (46.9) | 14 (51.9) | **0.02** |
|  | Inability to titrate fluid removal | 3 (2.7) | 3 (7.3) | 15 (4.9) | 0 | 0.39 |
|  | Unavailability of adequately trained nursing staff | 13 (11.8) | 3 (7.3) | 17 (5.5) | 4 (14.8) | 0.085 |
|  | Unavailability of dialysis machines | 9 (8.2) | 4 (9.8) | 12 (3.9) | 4 (14.8) | **0.04** |
|  | Cost associated with treatment | 4 (3.6) | 2 (4.9) | 15 (4.9) | 2 (7.4) | 0.86 |
|  |  |  |  |  |  |  |
| I believe early fluid removal is beneficial | |  |  |  |  |  |
|  | Strongly agree | 35 (31.8) | 13 (31.7) | 106 (34.5) | 5 (18.5) | 0.45 |
|  | Agree | 44 (40.0) | 15 (36.6) | 125 (40.7) | 11 (40.7) |  |
|  | Somewhat agree | 19 (17.3) | 8 (19.5) | 53 (17.3) | 6 (22.2) |  |
|  | Neither agree nor disagree | 9 (8.2) | 2 (4.9) | 18 (5.9) | 5 (18.5) |  |
|  | Somewhat disagree | 2 (1.8) | 2 (4.9) | 4 (1.3) | 0 |  |
|  | Disagree | 1 (0.9) | 1 (2.4) | 1 (0.3) | 0 |  |
|  | Strongly disagree | 0 | 0 | 0 | 0 |  |
|  |  |  |  |  |  |  |
| I believe a protocol-based fluid removal strategy would be beneficial | |  |  |  |  |  |
|  | Strongly agree | 37 (33.6) | 9 (22.0) | 71 (23.1) | 6 (22.2) | 0.13 |
|  | Agree | 24 (21.8) | 13 (31.7) | 103 (33.6) | 8 (29.6) |  |
|  | Somewhat agree | 24 (21.8) | 8 (19.5) | 60 (19.5) | 11 (40.7) |  |
|  | Neither agree nor disagree | 16 (14.6) | 4 (9.8) | 30 (9.8) | 2 (7.4) |  |
|  | Somewhat disagree | 5 (4.6) | 4 (9.8) | 19 (6.2) | 0 |  |
|  | Disagree | 3 (2.7) | 1 (2.4) | 18 (5.9) | 0 |  |
|  | Strongly disagree | 1 (0.9) | 2 (4.9) | 6 (2.0) | 0 |  |
|  |  |  |  |  |  |  |
| I would enroll my patient in a clinical trial comparing protocol-based versus usual care | |  |  |  |  |  |
|  | Strongly agree | 29 (26.6) | 9 (22.0) | 84 (27.4) | 5 (18.5) | 0.22 |
|  | Agree | 38 (34.9) | 16 (39.0) | 133 (43.3) | 8 (29.6) |  |
|  | Somewhat agree | 22 (20.2) | 8 (19.5) | 36 (11.7) | 6 (22.2) |  |
|  | Neither agree nor disagree | 15 (13.8) | 5 (12.2) | 33 (10.8) | 8 (29.6) |  |
|  | Somewhat disagree | 1 (0.9) | 1 (2.4) | 9 (2.9) | 0 |  |
|  | Disagree | 4 (3.7) | 1 (2.4) | 10 (3.3) | 0 |  |
|  | Strongly disagree | 0 | 1 (2.4) | 2 (0.7) | 0 |  |

Abbreviations: CRRT, continuous renal replacement therapy; HR, heart rate; BP, blood pressure; CVP, central venous pressure; PPV, pulse pressure variation; IHD, intermittent haemodialysis; IQR, interquartile range; UF^net^, net ultrafiltration; CT, computed tomography

^a^ Practitioners included intensivists, nephrologists, intensivists and nephrologists, and advanced practice providers. ICU and dialysis nurses were excluded from these questions.

**Supplementary Figure S2** Modalities of RRT use in each type of hospital

**
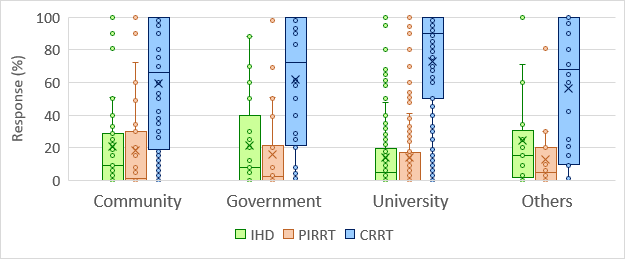
**

Abbreviations; IHD, intermittent hemodialysis; PIRRT, prolonged intermittent renal replacement therapy; CRRT, continuous renal replacement therapy

**Supplementary Table S3** Comparison by seven top respondent countries

| **Characteristic** | | **No. (%)** | | | | | | |  |
| --- | --- | --- | --- | --- | --- | --- | --- | --- | --- |
|  |  | **UK**  **(n=181)** | **Italy**  **(n=78)** | **Spain**  **(n=31)** | **Greece**  **(n=24)** | **France**  **(n=22)** | **Portugal**  **(n=21)** | **Germany**  **(n=17)** | **P Value** |
| Occupation | |  |  |  |  |  |  |  |  |
| Physicians | | **101 (55.8)** | 67 (87.2) | 31 (100) | 24 (100) | 22 (100) | 19 (90.5) | 17 (100) | <0.001 |
| Nurses/nurse practitioners | | 80 (44.2) | 10 (12.8) | 0 | 0 | 0 | 2 (9.5) | 0 |  |
|  |  |  |  |  |  |  |  |  |  |
| Years of practice, median (IQR) | | 15 (9-22) | 15 (7-24) | 15  (7.3-20.3) | 20.1  (14.8-22.1) | 20.5  (11-25.3) | 13.5 (10.1-19) | 13.2  (12-25.3) | 0.23 |
| Type of hospital | |  |  |  |  |  |  |  |  |
| University-based | | **126 (69.6)** | **48 (61.5)** | **25 (80.7)** | 4 (16.7) | **12 (54.6)** | **12 (57.1)** | 7 (41.2) | <0.001 |
| Community-based | | 14 (7.7) | 28 (35.9) | 3 (9.7) | **10 (41.7)** | 7 (31.8) | 5 (23.8) | **10 (58.8)** |  |
| Government | | 27 (14.9) | 0 | 2 (6.5) | 5 (20.8) | 1 (4.6) | 3 (14.3) | 0 |  |
| Others | | 14 (7.7) | 2 (2.6) | 1 (3.2) | 5 (20.8) | 2 (9.1) | 1 (4.8) | 0 |  |
|  | |  |  |  |  |  |  |  |  |
| Maximum dose of loop diuretic prescribed (furosemide equivalent) ^a^, mgs/day (n=289) | |  |  |  |  |  |  |  |  |
|  | <100 | 17 (16.4) | 4 (5.7) | 1 (3.2) | 1 (4.2) | 4 (18.2) | 2 (9.5) | 0 | <0.001 |
|  | 100 – 250 | **54 (51.9)** | 12 (17.1) | 4 (12.9) | 5 (20.8) | 3 (13.6) | **10 (47.6)** | **6 (35.3)** |  |
|  | 251 – 500 | 15 (14.4) | 17 (24.3) | 4 (12.9) | 5 (20.8) | 2 (9.1) | 4 (19.1) | 3 (17.7) |  |
|  | 501 – 750 | 0 | 6 (8.6) | 6 (19.4) | 4 (16.7) | 1 (4.6) | 2 (9.5) | 1 (5.9) |  |
|  | 751 – 1000 | 1 (1.0) | **21 (30.0)** | **10 (32.3)** | **7 (29.2)** | **7 (31.8)** | 2 (9.5) | 4 (23.5) |  |
|  | >1000 | 1 (1.0) | 10 (14.3) | 4 (12.9) | 2 (8.3) | 5 (22.7) | 0 | 0 |  |
|  | Others | 16 (15.3) | 0 | 2 (6.5) | 0 | 0 | 1 (4.8) | 3 (17.6) |  |
|  |  |  |  |  |  |  |  |  |  |
| Criteria used for initiation of UF^NET a^ (n=289) | |  |  |  |  |  |  |  |  |
|  | Persistent oliguria/anuria (urine output <0.5mL/kg/hour for ≥12 hours) | 26 (25.0) | **48 (68.6)** | **23 (74.2)** | **13 (54.2)** | 6 (27.3) | 6 (28.6) | **10 (58.5)** | <0.001 |
|  | Severe hypoxemia (PaO_2_/FiO_2_ ratio <150) | 11 (10.6) | 4 (5.7) | 3 (9.7) | 2 (8.3) | 5 (22.7) | 5 (23.8) | 0 |  |
|  | Pulmonary edema with or without hypoxemia | **34 (32.7)** | 3 (4.3) | 1 (3.2) | 3 (12.5) | 6 (27.3) | 1 (4.8) | 3 (17.7) |  |
|  | Cumulative fluid balance (>1000 mL) | 10 (9.6) | 2 (2.9) | 1 (3.2) | 0 | 1 (4.6) | 3 (14.3) | 0 |  |
|  | Fluid overload >10% of body weight | 4 (3.9) | 3 (4.3) | 0 | 1 (4.2) | 3 (13.6) | 2 (9.5) | 3 (17.7) |  |
|  | Ongoing need for fluids in the presence of oliguria | 6 (5.8) | 5 (7.1) | 2 (6.5) | 4 (16.7) | 0 | 0 | 0 |  |
|  | I do not make the decision/others | 13 (12.5) | 5 (7.1) | 1 (3.2) | 1 (4.2) | 1 (4.6) | 4 (19.1) | 1 (5.9) |  |
|  |  |  |  |  |  |  |  |  |  |
| Criteria used for prescription of UF^NET a^ (n=289) | |  |  |  |  |  |  |  |  |
|  | 24-hour fluid balance | 17 (16.4) | 15 (21.4) | 7 (22.6) | 4 (16.7) | 3 (13.6) | 5 (23.8) | 3 (17.7) | 0.19 |
|  | Cumulative fluid balance | 27 (26.0) | 14 (20.0) | 6 (19.4) | 4 (16.7) | 6 (27.3) | 1 (23.8) | 3 (17.7) |  |
|  | Weight gain | 3 (2.9) | 8 (11.4) | 2 (6.5) | 0 | 6 (27.3) | 0 | 1 (5.9) |  |
|  | Radiographic features of fluid overload | 4 (3.9) | 1 (1.4) | 0 | 0 | 0 | 0 | 0 |  |
|  | Hemodynamic status (HR, BP, CVP, PPV, dose of vasopressors) | 46 (44.2) | 28 (40.0) | 13 (41.9) | 14 (58.3) | 7 (31.8) | 14 (66.7) | 8 (47.1) |  |
|  | Volume of anticipated fluid use in the next 24 hours | 0 | 1 (1.4) | 2 (6.5) | 1 (4.2) | 0 | 0 | 0 |  |
|  | Others | 7 (6.7) | 3 (4.3) | 1 (3.2) | 1 (4.2) | 0 | 0 | 0 |  |
|  |  |  |  |  |  |  |  |  |  |
| IHD use, median (IQR) | |  |  |  |  |  |  |  |  |
|  | Percent use last month | 2.0 (0-27) | 9 (1-20) | 10 (1-15) | 12.5 (0-30) | 25 (6-50) | 10 (5-22) | 9 (3-50) | 0.13 |
|  | Typical prescription, mL per session | 1900 (1500-2500) | 2000 (1500-2100) | 2000 (1500-3000) | 2000 (1800-3000) | 2800 (2100-3000) | 2100 (2000-3000) | 2000 (2000-2500) | 0.07 |
|  |  |  |  |  |  |  |  |  |  |
| Slow forms of IHD use, median (IQR) | |  |  |  |  |  |  |  |  |
|  | Percent use last month | 0 (0-10) | 5 (0-20) | 3 (0-15) | 1 (0-6) | 8  (0-49.5) | **25**  **(10-41)** | 9 (0-55) | 0.001 |
|  | Typical prescription, mL per session | 1300 (1000-2000) | 2000 (900-2500) | 2000 (1500-2000) | 600  (0-1000) | **3500 (2000-4000)** | 2000 (2000-2500) | 2000 (1000-2000) | 0.03 |
|  |  |  |  |  |  |  |  |  |  |
| Percent of assessment of prescription-to-delivered UF^NET^, median (IQR) | | 88 (31-100) | 33 (15-87) | 30 (16-74) | 49.5 (22.5-87.5) | 85.5 (70-100) | **100 (91-100)** | **100 (92-100)** | 0.0002 |
|  |  |  |  |  |  |  |  |  |  |
| CRRT use, median (IQR) | |  |  |  |  |  |  |  |  |
|  | Percent use in the last month | **98 (70-100)** | 80 (15-100) | 84 (20-98) | 40 (7-90) | 50 (20-81) | 42 (12-80) | 68 (45-95.5) | 0.0001 |
|  | Initial UF^NET^ rate for hemodynamically stable patient, mL per hour | 122 (100-200) | 146.5 (100-200) | 151 (103-197) | 155 (101-200) | 193.5 (125.5-223) | 152.5 (100.5-200.5) | 101 (94-201) | 0.28 |
|  | Maximal UF^NET^ rate for hemodynamically stable patient, mL per hour | **300 (246-400)** | 221 (197-300) | 253 (200-313) | **300 (250-351)** | **200.5 (250.5-372.5)** | 251 (200-302) | 251 (225-392) | 0.0005 |
|  | UF^NET^ rate for hemodynamically unstable patient, mL per hour | 80 (49-101) | 98 (51-101) | 100 (59-107) | 100 (68-120) | 71 (49-116) | 100 (51-149) | 65 (0-100) | 0.17 |
|  |  |  |  |  |  |  |  |  |  |
| Method used to achieve UF^NET^ using CRRT, No. (%) (n=359) | |  |  |  |  |  |  |  |  |
|  | By varying ultrafiltration rate only | **84 (47.7)** | 15 (20.0) | 6 (20.0) | 7 (31.8) | 9 (45.0) | 0 | 0 | <0.001 |
|  | By varying replacement fluid rate only | 14 (8.0) | 3 (4.0) | 5 (16.7) | 0 | 4 (20.0) | 0 | 0 |  |
|  | By varying both ultrafiltration and replacement fluid rate | 53 (30.1) | **47 (62.7)** | **18 (60.0)** | **14 (63.6)** | 6 (30.0) | 7 (33.3) | 12 (80.0) |  |
|  | I do not know. | 20 (11.4) | 8 (10.7) | 1 (3.3) | 1 (4.6) | 0 (0.0) | 0 (0.0) | 0 (0.0) |  |
|  | I do not prescribe UF. | 5 (2.8) | 2 (2.7) | 0 (0.0) | 0 (0.0) | 1 (5.0) | 0 (0.0) | 0 (0.0) |  |
|  |  |  |  |  |  |  |  |  |  |
| How frequently do you check net fluid balance during CRRT? No. (%) (n=359) | |  |  |  |  |  |  |  |  |
|  | 1 hour | **89 (50.6)** | 4 (5.3) | 4 (13.3) | 1 (4.6) | 1 (5.0) | 1 (4.8) | 5 (33.3) | <0.001 |
|  | 2 hours | 3 (1.7) | 6 (8.0) | 2 (6.7) | 1 (4.6) | 0 | 0 | 0 |  |
|  | 4 hours | 17 (9.7) | 3 (4.0) | 2 (6.7) | 4 (18.2) | 3 (15.0) | 3 (14.3) | 3 (20.0) |  |
|  | 6 hours | 15 (8.5) | 18 (24.0) | 5 (16.7) | 1 (4.6) | 3 (15.0) | 1 (4.8) | 1 (6.7) |  |
|  | 8 hours | 11 (6.3) | 6 (8.0) | 5 (16.7) | 10 (45.5) | 2 (10.0) | 9 (42.9) | 1 (6.7) |  |
|  | 12 hours | 16 (9.1) | 22 (29.3) | 2 (6.7) | 0 | 0 | 0 | 0 |  |
|  | 24 hours | 8 (4.6) | 10 (13.3) | 9 (30.0) | 2 (9.1) | 6 (30.0) | 2 (9.5) | 1 (6.7) |  |
|  | I do not check net fluid balance. | 17 (9.7) | 6 (8.0) | 1 (3.3) | 3 (13.6) | 0 (0.0) | 4 (19.1) | 1 (6.7) |  |
|  |  |  |  |  |  |  |  |  |  |
| Percentage of patients developing new hemodynamic instability during UF^NET^, median (IQR) | | 20 (10-29) | 15 (5-30) | 12 (5-25) | 30 (7-41) | 27.5 (20-41.5) | **11 (9-25)** | 20 (10-31.5) | 0.02 |
|  |  |  |  |  |  |  |  |  |  |
|  |  |  |  |  |  |  |  |  |  |
|  |  |  |  |  |  |  |  |  |  |
|  |  |  |  |  |  |  |  |  |  |
| Interventions performed for hemodynamic instability | |  |  |  |  |  |  |  |  |
|  | Decrease the rate of fluid removal | 134 (74.0) | 48 (61.5) | 22 (71.0) | 19 (79.2) | 14 (63.6) | 16 (76.2) | 13 (76.5) | 0.41 |
|  | Completely stop fluid removal | **91 (50.3)** | 6 (7.7) | 7 (22.6) | 5 (20.8) | 8 (36.4) | 6 (28.6) | 4 (23.5) | <0.001 |
|  | Make no changes to fluid removal rate | 9 (5.0) | 2 (2.6) | 0 | 0 | 1 (4.6) | 0 | 0 | 0.53 |
|  | Administer a fluid bolus | **89 (49.2)** | 23 (29.5) | 8 (25.8) | 11 (45.8) | 5 (22.7) | 4 (19.1) | 7 (41.2) | 0.003 |
|  | Start or increase the dose of a vasopressor | 92 (50.8) | 38 (48.7) | 13 (41.9) | 15 (62.5) | 11 (50) | 11 (52.4) | 11 (64.7) | 0.71 |
|  | Switch to alternative modality | 5 (2.8) | 1 (1.3) | 1 (3.2) | 1 (4.2) | 2 (9.1) | 2 (9.5) | 2 (11.8) | 0.20 |
|  | Administer albumin or mannitol bolus | 24 (13.3) | 8 (10.3) | 2 (6.5) | 0 | 3 (13.6) | 0 | 1 (5.9) | 0.23 |
|  |  |  |  |  |  |  |  |  |  |
| Perceived barriers to UF^NET^ | |  |  |  |  |  |  |  |  |
|  | Patient intolerance (*e.g.,* hypotension) | 150 (82.9) | 38 (48.7) | 21 (67.7) | 19 (79.2) | 16 (72.7) | 18 (85.7) | **15 (88.2)** | <0.001 |
|  | Under prescription | 27 (14.9) | 8 (10.3) | 5 (16.1) | 5 (20.8) | 6 (27.3) | 3 (14.3) | 2 (11.8) | 0.57 |
|  | Frequent interruptions (*e.g.,* trip to CT scan, operating room, filter clotting, catheter malfunction) | **106 (58.6)** | 33 (42.3) | 15 (48.4) | 9 (37.5) | 7 (31.8) | 8 (38.1) | 4 (23.5) | 0.008 |
|  | Inability to titrate fluid removal | 11 (6.1) | 3 (3.9) | 3 (9.7) | 1 (4.2) | 1 (4.6) | 0 | 0 | 0.67 |
|  | Unavailability of adequately trained nursing staff | 11 (6.1) | 10 (12.8) | 3 (9.7) | 2 (8.3) | 3 (13.6) | 1 (4.8) | 1 (5.9) | 0.59 |
|  | Unavailability of dialysis machines | 12 (6.6) | 2 (2.6) | 2 (6.5) | 3 (12.5) | 2 (9.1) | 3 (14.3) | 3 (17.7) | 0.23 |
|  | Cost associated with treatment | 5 (2.8) | 5 (6.4) | 1 (3.2) | 2 (8.3) | 0 | 0 | 1 (5.9) | 0.50 |
|  |  |  |  |  |  |  |  |  |  |
| I believe early fluid removal is beneficial | |  |  |  |  |  |  |  |  |
|  | Strongly agree | 51 (28.2) | 26 (33.3) | 13 (41.9) | 5 (20.8) | 4 (18.2) | 9 (42.9) | 10 (58.8) | 0.49 |
|  | Agree | 71 (39.2) | 32 (41.0) | 12 (38.7) | 13 (54.2) | 14 (63.6) | 7 (33.3) | 4 (23.5) |  |
|  | Somewhat agree | 41 (22.7) | 12 (15.4) | 5 (16.1) | 5 (20.8) | 2 (9.1) | 3 (14.3) | 2 (11.8) |  |
|  | Neither agree nor disagree | 16 (8.8) | 4 (5.1) | 1 (3.2) | 1 (4.2) | 1 (4.6) | 1 (4.8) | 1 (5.9) |  |
|  | Somewhat disagree | 1 (0.6) | 3 (3.9) | 0 | 0 | 1 (4.6) | 1 (4.8) | 0 |  |
|  | Disagree | 1 (0.6) | 1 (1.3) | 0 | 0 | 0 | 0 | 0 |  |
|  | Strongly disagree | 0 | 0 | 0 | 0 | 0 | 0 | 0 |  |
|  |  |  |  |  |  |  |  |  |  |
| I believe a protocol-based fluid removal strategy would be beneficial | |  |  |  |  |  |  |  |  |
|  | Strongly agree | 24 (13.3) | 31 (39.7) | 9 (29.0) | 9 (37.5) | 7 (31.8) | 6 (28.6) | 5 (29.4) | <0.001 |
|  | Agree | 43 (23.8) | 27 (34.6) | 16 (51.6) | 9 (37.5) | 9 (40.9) | 6 (28.6) | 5 (29.4) |  |
|  | Somewhat agree | 47 (26.0) | 16 (20.5) | 5 (16.1) | 6 (25.0) | 4 (18.2) | 5 (23.8) | 4 (23.5) |  |
|  | Neither agree nor disagree | 25 (13.8) | 2 (2.6) | 0 | 0 | 2 (9.1) | 2 (9.5) | 1 (5.9) |  |
|  | Somewhat disagree | 20 (11.1) | 1 (1.3) | 1 (3.2) | 0 | 0 | 0 | 2 (11.8) |  |
|  | Disagree | 15 (8.3) | 1 (1.3) | 0 | 0 | 0 | 2 (9.5) | 0 |  |
|  | Strongly disagree | 7 (3.9) | 0 | 0 | 0 | 0 | 0 | 0 |  |
|  |  |  |  |  |  |  |  |  |  |
| I would enroll my patient in a clinical trial comparing protocol-based versus usual care | |  |  |  |  |  |  |  |  |
|  | Strongly agree | 33 (18.2) | 27 (35.1) | 7 (22.6) | 8 (33.3) | 11 (50.0) | 9 (42.9) | 0 | 0.04 |
|  | Agree | 76 (42.0) | 34 (44.2) | 17 (54.8) | 6 (25.0) | 6 (27.3) | 10 (47.6) | 7 (41.2) |  |
|  | Somewhat agree | 25 (13.8) | 8 (10.4) | 3 (9.7) | 5 (20.8) | 3 (13.6) | 2 (9.5) | 6 (35.3) |  |
|  | Neither agree nor disagree | 31 (17.1) | 5 (6.5) | 3 (9.7) | 5 (20.8) | 2 (9.1) | 0 | 3 (17.7) |  |
|  | Somewhat disagree | 5 (2.8) | 1 (1.3) | 1 (3.2) | 0 | 0 | 0 | 1 (5.9) |  |
|  | Disagree | 9 (5.0) | 2 (2.6) | 0 | 0 | 0 | 0 | 0 |  |
|  | Strongly disagree | 2 (1.1) | 0 | 0 | 0 | 0 | 0 | 0 |  |

Abbreviations: CRRT, continuous renal replacement therapy; HR, heart rate; BP, blood pressure; CVP, central venous pressure; PPV, pulse pressure variation; IHD, intermittent haemodialysis; IQR, interquartile range; UF^net^, net ultrafiltration; CT, computed tomography

^a^ Practitioners included intensivists, nephrologists, intensivists and nephrologists, and advanced practice providers. ICU and dialysis nurses were excluded from these questions.

**Supplementary Table S4** Net Ultrafiltration Rates by Country

| **Country** | **No. of observations** | **Median (IQR)** | | |
| --- | --- | --- | --- | --- |
|  |  | **Hemodynamically**  **Stable Patients** | | **Hemodynamically Unstable Patients** |
|  |  | **Initial**  **UF^NET^ Rate**  **(mL/h)** | **Maximal**  **UF^NET^ Rate**  **(mL/h)** | **Typical**  **UF^NET^ Rate**  **(mL/h)** |
| Austria | 3 | 151.0 (100.0 – 200.0) | 400.0 (100.0 – 400.0) | 100.0 (25.0 – 100.0) |
| Belgium | 13 | 107.0 (100.0 – 198.0) | **452.0 (300.0 – 712.0)** | 51.0 (27.5 – 115.0) |
| Bulgaria | 4 | 177.0 (126.5 – 227.0) | 326.0 (206.5 – 351.5) | 99.5 (96.0 – 209.5) |
| Croatia | 1 | 179.0 (179.0 – 179.0) | 301.0 (301.0 – 301.0) | 50.0 (50.0 – 50.0) |
| Czech Republic | 1 | 153.0 (153.0 – 153.0) | 314.0 (314.0 – 314.0) | 110.0 (110.0 – 110.0) |
| Denmark | 15 | 152.0 (100.0 – 203.0) | 302.0 (300.0 – 397.0) | 100.0 (76.0 – 192.0) |
| Estonia | 3 | 151.0 (100.0 – 200.0) | 400.0 (300.0 – 500.0) | **151.0 (50.0 – 500.0**) |
| Finland | 1 | 103.0 (103.0 – 103.0) | 200.0 (200.0 – 200.0) | 49.0 (49.0 – 49.0) |
| France | 20 | **193.5 (125.5 – 223.0)** | 300.5 (250.5 – 372.5) | 71.0 (49.0 – 116.0) |
| Germany | 15 | 101.0 (94.0 – 201.0) | 251.0 (225.0 – 392.0) | 65.0 (0 – 100.0) |
| Greece | 22 | 155.0 (101.0 – 200.0) | 300.0 (250.0 – 351.0) | 100.0 (68.0 – 120.0) |
| Hungary | 2 | 110.0 (100.0 – 120.0) | 231.0 (161.0 – 301.0) | 64.5 (49.0 – 80.0) |
| Iceland | 2 | 150.0 (100.0 – 200.0) | 353.0 (200.0 – 506.0) | 75.5 (51.0 – 100.0) |
| Ireland | 4 | 125.5 (100.0 – 164.0) | 203. 5 (200.0 – 230.5) | 50.0 (36.0 – 90.5) |
| Italy | 74 | 146.5 (100.0 – 200.0) | 221.0 (197.0 – 300.0) | 98.0 (51.0 – 101.0) |
| Lithuania | 2 | 150.0 (100.0 – 200.0) | **199.5 (151.0 – 248.0)** | 90.0 (0 – 180.0) |
| Malta | 1 | 155.0 (155.0 – 155.0) | 232.0 (232.0 – 232.0) | 126.0 (126.0 – 126.0) |
| Netherlands | 7 | **51.0 (50.0 – 92.0)** | 249.0 (190.0 – 300.0) | **10.0 (0 – 51.0)** |
| Norway | 5 | 149.0 (100.0 – 152.0) | 300.0 (252.0 – 311.0) | 100.0 (52.0 – 100.0) |
| Poland | 10 | 174.5 (107.0 – 200.0) | 276.5 (200.0 – 400.0) | 125.0 (94.0 – 151.0) |
| Portugal | 19 | 152.5 (100.5 – 200.5) | 251.0 (200.0 – 302.0) | 100.0 (51.0 – 149.0) |
| Romania | 6 | 173.0 (100.0 – 300.0) | 401.0 (4000 – 503.0) | 103.0 (100.0 – 258.0) |
| Serbia | 4 | 151.0 (51.0 – 300.0) | 280.0 (251.5 – 654.0) | 28.0 (0 – 102.5) |
| Slovakia | 2 | 160.0 (120.0 – 200.0) | 225.5 (151.0 – 300.0) | 90.0 (80.0 – 100.0) |
| Spain | 28 | 151.0 (103.0 – 197.0) | 253.0 (200.0 – 313.0) | 100.0 (59.0 – 107.0) |
| Sweden | 9 | 124.0 (100.0 – 177.0) | 399.0 (275.5 – 500.0) | 103.0 (100.0 – 151.0) |
| Switzerland | 6 | 100.0 (51.0 – 101.0) | 225.0 (200.0 – 351.0) | 50.0 (5.0 – 101.0) |
| Turkey | 14 | 148.0 (100.0 – 200.0) | 200.0 (187.5 – 253.0) | 77.5 (51.0 – 100.0) |
| Ukraine | 1 | - | - | 103.0 (103.0 – 103.0) |
| United Kingdom | 175 | 122.0 (100.0 – 200.0) | 300.0 (246.0 – 400.0) | 80.0 (49.0 – 101.0) |

# Supplementary Table S5 Thematic Analysis of Comments by Practitioner Type

|  | | | **No. (%)** | | |
| --- | --- | --- | --- | --- | --- |
| **Theme or Subtheme** | | | **All**  **Comments**  **(n=156)** | **Physician**  **Comments**  **(n=133)** | **Nurse**  **Comments**  **(n=23)** |
| Tools to achieve UF^NET^ | | |  |  |  |
|  | Functional hemodynamic monitoring | | 18 (11.5%) | 18 (13.5%) | 0 |
|  | Biomarker | | 1 (0.6%) | 1 (0.8%) | 0 |
|  | Use of protocol | | 6 (3.8%) | 3 (2.3%) | 3 (13.0%) |
|  | Use of precision medicine | | 8 (5.1%) | 6 (4.5%) | 2 (8.7%) |
|  | Use of vasopressors | | 3 (1.9%) | 3 (2.3%) | 0 |
|  | Prescription-related factors | | 11 (7.1%) | 10 (7.5%) | 1 (4.3%) |
| Organizational factors | | |  |  |  |
|  | Resources | | 3 (1.9%) | 2 (1.5%) | 1 (4.3%) |
|  | Practice characteristics | | 1 (0.6%) | 0 | 1 (4.3%) |
|  | Practice variation | | 3 (1.9%) | 0 | 3 (13.0%) |
| Clinician-related factors | | |  |  |  |
|  | Education/Training | | 4 (2.6%) | 2 (1.5%) | 2 (8.7%) |
|  | Experience | | 2 (1.3%) | 2 (1.5%) | 2 (8.7%) |
|  | Communication | | 1 (0.6%) | 1 (0.8%) | 0 |
| Patient-related factors | | |  |  |  |
|  | Underlying cause of FO | | 9 (5.8%) | 9 (6.8%) | 0 |
|  | Assessment of fluid overload | | 12 (7.7%) | 12 (9.0%) | 0 |
|  | Comorbidities | | 14 (9.0%) | 13 (9.8%) | 1 (4.3%) |
|  | Intravascular volume assessment | | 8 (5.1%) | 8 (6.0%) | 0 |
|  | Fluid management | | 6 (3.8%) | 4 (3.0%) | 2 (8.7%) |
|  | Monitoring | | 4 (2.6%) | 3 (2.3%) | 1 (4.3%) |
|  | Oliguria treatment | | 2 (1.3%) | 2 (1.5%) | 0 |
|  | Complication management | | 6 (3.8%) | 6 (4.5%) | 0 |
| RRT related factors | | |  |  |  |
|  | | Anticoagulation | 3 (1.9%) | 3 (2.3%) | 0 |
|  | | Treatment interruption | 3 (1.9%) | 2 (1.5%) | 1 (4.3%) |
|  | | Timing of initiation and cessation of UF^NET^ | 11 (7.1%) | 10 (7.5%) | 1 (4.3%) |
|  | | Modality | 5 (3.2%) | 5 (3.8%) | 0 |
| Miscellaneous | | | 10 (6.4%) | 8 (6.0%) | 2 (8.7%) |

Thirteen patients had 2 responses; Two patients had 1 response; One patient had four responses.

Abbreviations: RRT, renal replacement therapy; FO, fluid overload; UF^net^, net ultrafiltration

# Supplementary Table S6 Examples of Comments Amenable to Research and Quality Improvement Interventions

| **Theme** | **Comments Amenable to Research** | **Comments Amenable to Quality Improvement** |
| --- | --- | --- |
| Vasopressor use during net ultrafiltration | “Earlier use of vasopressors in sepsis” – Intensivist  “Norepinephrine dose before starting CRRT” - Intensivist | “Avoiding excess fluid administration even if vasopressor dosage must be increased” – Intensivist |
| Practice variation | “Clear guidance for recommended UF rates would be extremely beneficial, there is nothing that I have seen in my time within critical care to guide nurses on fluid removal rates. I feel it is very variable between nurses, for example, one nurse may be happy with removing 350-400 mL/hr with a patient on 0.19 mcgs of noradrenaline and a slightly elevated but static lactate with this increase in UF, whereas another nurse may not be comfortable” – ICU nurse | “Changes of consultant occasionally leading to extreme changes to fluid balance i.e. replacement of large amounts of fluid and next day aggressively removing fluid. Perhaps a more consistent approach would result in less hemodynamic instability and better fluid management overall.” – ICU nurse |
| Treatment interruption |  | “When prescribing large fluid removal rates, the resulting rise in filtration fraction too often goes unnoticed and addressed, leading to earlier filter clogging and all that follows.” – Intensivist  “Barrier to treatment: vascular access especially in obese patients” - Intensivist |
| Timing | “Criteria for discontinuation of CRRT” – Intensivist  “Is not important if the patient may be wet or dry but when we can do it.” – Intensivist |  |
| Resource |  | “I would do a qualitative study looking at nurse or renal technician anxiety first. If you don't factor this in your protocol won’t get delivered” – Intensivist |
| Education |  | “Continuous RRT education for junior doctors & nurses. Ignorance and disagreements are key barriers in fluid management, in addition to misinterpretation of protocol” – ICU nurse |
| Precision Medicine |  | “I think the key factor in all these things is clinical findings and the way the patient behaves. I think fluid balance needs to be titrated to the individual patients’ needs.” - Intensivist |
| Protocol-based treatment | “I would consider an echo as part of the protocol” – Intensivist  “Although I feel a protocol-based plan may not work in a person-centred approach, continuity with medical staff is paramount.” – ICU nurse |  |
| Intravascular volume assessment | “Measure of plasma refill rate. This is probably an important factor that we do not measure accurately in ICU patients” – Intensivist  “Colloid osmotic pressure/ albumin concentration helps guide and manage rate of fluid loss” - Intensivist |  |
| Prescription of fluid removal | “I would not want to enrol patients in any trial where fluid removal was ongoing in the face of hemodynamic instability associated with all the clinical signs of intravascular depletion and prescription of vasopressors” – Intensivist  “I would be more aggressive with fluid removal if it was evidence based.” – Intensivist  “Renal, lung and heart echography use for initiation, titration and weaning RRT” – Intensivist  “The criteria for fluid removal should be more aggressive if the patient is hemodynamically stable.” – Intensivist  “Prioritize the factors used to stop/decrease fluid removal” – Intensivist and Nephrologist  “Clear parameters for what to do is haemodynamic instability occurs, a fluid balance aim to achieve by a particular time and if other parameters should be watched (e.g., FiO2 increase)” – ICU nurse | “The amount of prescribed fluid removal either by IHD or by SLED or CRRT depends on the overweight, positive fluid balance AND the overall ongoing fluid administration due to drugs delivery, nutrition also. You do consider this parameter to prescribe the net volume of fluid removal” - Intensivist |
| Hemodynamic monitoring | “What hemodynamic monitoring should be used before to stop the remove of fluid” – Intensivist | “As we have many cardiac surgery and other cardiologic patients it is important to check that cardiac output Is taken into account and cardiac failure treated before and while applying renal therapy” - Intensivist |
| Oliguria management | “Use of diuretics during CVVHDF for the assessment of spontaneous diuresis” - Intensivist |  |
| Assessment of fluid overload | “How to diagnose and measure fluid overload?” – Intensivist  “I think patient weight/weight change is a better marker of cumulative fluid balance status than the in/out balances that are commonly used.” – Intensivist | “I think that fluid overload is somewhat difficult to diagnose; many patients have too much fluid "on board" without meeting clear criteria of physiological derangements; many intensivists are reluctant to initiate RRT, as they want to try diuretics first; I am very much in favour of initiation RRT (either CRRT or SLED) in accordance with KDIGO criteria of AKI; RRT should in my opinion be initiated when reaching KDIGO grade 3.” – Intensivist |
| Training and experience |  | “I think a limiting factor in achieving fluid balance targets/adequate fluid removal is often due to inexperience of nursing staff caring for patients receiving intermittent or continuous fluid removal.” – ICU nurse |
